# Designing an Epitope-Based Peptide Vaccine Derived from RNA-Dependent RNA Polymerase (RdRp) against Dengue Virus Serotype 2

**DOI:** 10.3390/vaccines10101734

**Published:** 2022-10-17

**Authors:** Irma F. Wahongan, Elly J. Suoth, Fatimawali, Saad Alhumaid, Hawra Albayat, Mohammed Aljeldah, Basim R. Al Shammari, Mutaib M. Mashraqi, Ahmad A. Alshehri, Tarek Sulaiman, Safaa A. Turkistani, Ameen S. S. Alwashmi, Mohammed Garout, Trina Ekawati Tallei, Ali A. Rabaan

**Affiliations:** 1 Pharmacy Study Program, Faculty of Mathematics and Natural Sciences, Sam Ratulangi University, Manado 95115, North Sulawesi, Indonesia; 2 Administration of Pharmaceutical Care, Al-Ahsa Health Cluster, Ministry of Health, Al-Ahsa 31982, Saudi Arabia; 3 Infectious Disease Department, King Saud Medical City, Riyadh 7790, Saudi Arabia; 4 Department of Clinical Laboratory Sciences, College of Applied Medical Sciences, University of Hafr Al Batin, Hafr Al Batin 39831, Saudi Arabia; 5 Department of Clinical Laboratory Sciences, College of Applied Medical Sciences, Najran University, Najran 61441, Saudi Arabia; 6 Infectious Diseases Section, Medical Specialties Department, King Fahad Medical City, Riyadh 12231, Saudi Arabia; 7 Fakeeh College for Medical Science, Jeddah 21134, Saudi Arabia; 8 Department of Medical Laboratories, College of Applied Medical Sciences, Qassim University, Buraydah 51452, Saudi Arabia; 9 Department of Community Medicine and Health Care for Pilgrims, Faculty of Medicine, Umm Al-Qura University, Makkah 21955, Saudi Arabia; 10 Department of Biology, Faculty of Mathematics and Natural Sciences, Sam Ratulangi University, Manado 95115, North Sulawesi, Indonesia; 11 Molecular Diagnostic Laboratory, Johns Hopkins Aramco Healthcare, Dhahran 31311, Saudi Arabia; 12 College of Medicine, Alfaisal University, Riyadh 11533, Saudi Arabia; 13 Department of Public Health and Nutrition, The University of Haripur, Haripur 22610, Pakistan

**Keywords:** dengue fever, DENV-2, RNA-dependent RNA polymerase, dendritic cells, NS5, vaccine

## Abstract

Dengue fever (DF) continues to be one of the tropical and subtropical health concerns. Its prevalence tends to increase in some places in these regions. This disease is caused by the dengue virus (DENV), which is transmitted through the mosquitoes *Aedes aegypti* and *A. albopictus*. The treatment of DF to date is only supportive and there is no definitive vaccine to prevent this disease. The non-structural DENV protein, RNA-dependent RNA Polymerase (RdRp), is involved in viral replication. The RdRp-derived peptides can be used in the construction of a universal dengue vaccine. These peptides can be utilized as epitopes to induce immunity. This study was an in silico evaluation of the affinity of the potential epitope for the universal dengue vaccine to dendritic cells and the bonds between the epitope and the dendritic cell receptor. The peptide sequence MGKREKKLGEFGKAKG generated from dengue virus subtype 2 (DENV-2) RdRp was antigenic, did not produce allergies, was non-toxic, and had no homology with the human genome. The potential epitope-based vaccine MGKREKKLGEFGKAKG binds stably to dendritic cell receptors with a binding free energy of −474,4 kcal/mol. This epitope is anticipated to induce an immunological response and has the potential to serve as a universal dengue virus vaccine candidate.

## 1. Introduction

Dengue fever (DF) is a vector-borne infectious disease that is transmitted by the dengue virus (DENV) through mosquito bites [1]. The prevalence of DF has increased 30-fold in the last 50 years, making it a global health concern. An estimated 390 million DENV infections occur each year, with 96 million exhibiting symptoms. Asia accounts for 70% of these cases [2]. The increase in dengue cases demands the development of a vaccine that can induce an immunological response to prevent infections, specifically the development of vaccinations that can induce an immunological response [3]. Until now, DF treatment has only been supportive with the aim of providing comfort to the patient and reducing the patient’s symptoms [4]. Efforts to reduce the high mortality rate caused by DENV require preventive measures. One of them is through vaccination. The development of a vaccine based on a peptide is one of the possible approaches to the problem. Peptide vaccines are made from in vitro synthesized peptides containing 8 to 30 amino acids, which are highly immunogenic and able to elicit an immune response [5,6,7].

DENV is a member of the genus *Flavivirus* of the family *Flaviviridae* [8]. This virus causes a wide range of diseases in humans, from the acute febrile illness dengue fever (DF) to life-threatening dengue hemorrhagic fever/dengue shock syndrome (DHF/DSS) [9,10]. Currently, only a small number of FDA-approved vaccines and antiviral treatments are available for the prevention or treatment of DENV infection. Dengvaxia, which is considered as a safe and effective dengue vaccine candidate, is currently undergoing clinical trials and is the only vaccine currently approved by the FDA [11]. 

The DENV comprises distinct serotypes [12], with serotype 2 (DENV-2) being the most prevalent and malignant of the four [13,14], especially in the tropics. In comparison to DENV-1 and DENV-4 cases, DENV-2 cases in Brazil had a higher percentage of severe dengue cases [14]. However, this does not rule out the possibility that the development of severe manifestations may involve host, epidemiological, and viral factors [15]. The virus encodes seven nonstructural proteins including NS1, NS2a, NS2b, NS3, NS4a, NS4b, and NS5 [16]. The non-structural protein NS5 of *Flavivirus* is about 900 amino acids long and comprises a methyltransferase domain at its N-terminus and an RNA-dependent RNA polymerase (RdRp) domain at its C-terminal end, which play a role in the viral replication process [17,18]. This protein can serve as promising antiviral development targets [19,20], in addition to its potential as antigen for a dengue vaccine formulation [21]. The generated peptides also may have the potential to be employed as vaccine candidates based on epitopes.

Utilizing immunoinformatics permits the design of potential peptide-based epitopes for a DENV vaccine [22,23]. The principle of designing a peptide-based epitope vaccine is to predict the interaction between RdRp DENV and dendritic cells [24]. This vaccine functions as an antigen that can be targeted to one of the antigen-presenting cells (APCs) to stimulate an immune response. [25]. Dendritic cells are immune system cells that control adaptive immune responses and can identify infections that activate T cells and B cells [26]. Antigens bound by dendritic cells will be ingested by the cytosol and cut into peptides, which will subsequently be presented in MHC for T cells and B cells, inducing a specific immune response that can be recognized by all organs of the body when utilized as vaccine candidates [26,27,28]. It is essential that epitope vaccines interact with dendritic cell receptors in order to elicit an immune response [29]. According to studies in mice, targeting antigens to dendritic cells (DCs) can elicit robust CD4+T cell responses [30]. Additionally, DCs can process antigens, such as peptides. Several studies involving DCs for vaccine development have been reported [31]. Furthermore, directing antigens to DCs, the primary antigen presentation cells and orchestrators of the adaptive immune response, can boost immunogenicity by activating T and B cells [26].

In order to observe the interactions between dendritic cells and epitope-based vaccination candidates, this research involves molecular docking and dynamics modeling experiments. The proposed vaccine is a peptide generated from RdRp DENV-2 that will serve as an epitope-based vaccine, as predicted by the Immune Epitope Database Analysis Resource (IEDB-AR). As DCs play a significant role in a variety of diseases, they have become excellent targets for the development of new strategies to prevent and treat these diseases [29]. In this study, DC is utilized in the development of a dengue vaccine for DENV-2. 

## 2. Materials and Methods

### 2.1. DENV-2 Sequences Alignment and Reconstruction of Phylogenetic Tree

The DENV-2 sequences were retrieved from NCBI (https://www.ncbi.nlm.nih.gov, accessed on 2 April 2022). Using the FASTA format, the NS5 region encoding RNA-dependent RNA polymerase was then aligned. The phylogenetic tree was reconstructed using MEGAX with the neighbor joining method and 1000 replicates of the bootstrap. 

### 2.2. B-Cell Linear Epitope Analysis

The DENV-2 sequence from Sumatra (Indonesia) with accession number BAD42415 was selected for linear B cell analysis using the Immune Epitope Database (IEDB) (http://tools.iedb.org/, accessed on 4 April 2022). The results of the linear epitope analysis for B cells are located in the B cell region or the prediction region.

### 2.3. Immunological Properties Analysis

Four types of tests are used to assess epitope immunological properties: antigenicity testing, allergenicity testing, toxicity testing, and homology predictions. Antigenicity prediction was performed via the Vaxijen v.2.0 web server (http://www.ddgpharmfac.net/vaxijen/VaxiJen/VaxiJen.html, accessed on 5 April 2022). The AllergenFP v.2.0 web server (http://ddg-pharmfac.net/AllergenFP/, accessed on 6 April 2022) was used to predict allergenicity. Toxicity prediction was carried out using the ToxinPred web server (http://crdd.osdd.net/raghava/toxinpred/, accessed on 8 April 2022). For the assessment of homology with human protein, predictions were made using the Basic Local Alignment Search Tool (BLAST) (https://blast.ncbi.nlm.nih.gov/Blast.cgi?PROGRAM=blastp&PAGE_TYPE=BlastSearch&LINK_LOC=blasthome, accessed on 10 April 2022) [32]. 

### 2.4. Receptor Preparation

The three-dimensional protein structure of the dendritic cell that acts as a receptor was downloaded from the protein data bank with PDB ID 3WBP. The Biovia Discovery Studio software was used to prepare the receptor. This preparation aims to obtain a structure that is devoid of water molecules, making molecular docking easier to accomplish. Polar hydrogen atoms were added to the receptor using Autodock Tools [33]. Dendritic cell protein structure quality was analyzed on the PROCHECK web server (https://saves.mbi.ucla.edu/, accessed on 11 April 2022) [34] and displayed in a Ramachandran plot [35]. 

### 2.5. Peptide Modeling and Molecular Docking

The selected epitope obtained based on the results of the analysis was then modeled using the PEP-FOLD web server (https://mobyle.rpbs.univ-paris-diderot.fr/cgi-bin/portal.py#forms::PEP-FOLD, accessed on 12 April 2022). The peptide epitope was subsequently docked with a prepared dendritic cell receptor. The process of molecular docking is performed on ClusPro web server designed specifically for protein–protein docking [36]. The results of molecular docking were visualized using the PyMOL software incorporated into the PDBsum web server (http://www.ebi.ac.uk/thornton-srv/databases/pdbsum/Generate.html, accessed on 15 April 2022) [37]. 

### 2.6. Molecular Dynamics Simulation Study

Simulations on the molecular dynamics (MD) of peptide and receptor interactions were carried out using a web-based CHARMM-GUI interface system [38,39,40] with a CHARMM36 force field [41]. All simulations were performed using the NAMD 2.13 package [42]. Utilizing the explicit solvation model TIP3P [43], periodic boundary conditions with dimensions of 106, 106, and 106 in x, y, and z, respectively, were defined. The system was then neutralized with 5Cl^−^ ions. The MD protocol involves minimization, equilibration, and production. The 2 femtoseconds time integration step was selected for all MD simulations. Equilibration was carried out in a canonical ensemble (NVT), while production took place in an isothermal–isobaric ensemble (NPT). Through the production of an MD of 100 ns, the pressure was set at 1 atm using a Langevin Nosé–Hoover piston barostat [44,45] with a Langevin piston decay of 0.05 ps and 0.1 ps period. The temperature was set at 298.15 K using a Langevin thermostat [46]. A distance limit of 12.0 Å was applied to short-range non-bonded interactions with a pair list distance of 16.0 Å, and the Lennard-Jones interaction smoothly truncated at 8.0 Å. Long-distance electrostatic interactions were treated using the particle mesh Ewald (PME) method [47,48], where a 1.0 Å grid spacing was used for all simulated cells. All covalent bonds involving hydrogen atoms are restricted using the SHAKE algorithm [49]. 

### 2.7. MM/GBSA Binding-Free Energy Calculation

Molecular mechanics/generalized born surface area (MM/GBSA) [50,51] is one method implemented in the MOLAICAL code [52] for calculating the relative binding-free energy that occurs when a peptide forms a complex with a protein receptor. The following formula was employed:

(1) $$\Delta G_{bind}=\Delta G_{RL}-\Delta G_{R}-\Delta G_{L}$$

which can be represented by different interaction contributions,

(2) $$\Delta G_{bind}=\Delta H-T\Delta S=\Delta E_{MM}+\Delta G_{Sol}-T\Delta S\;$$

where, the change in gas phase molecular mechanics ($\Delta E_{MM})$, Gibbs solvation energy ($\Delta G_{Sol})$, and conformational entropy (TΔS) is determined as follows: $\Delta E_{MM}$ is the sum of the changes in electrostatic energy $\left(\Delta E_{ele}\right)$, van der Waals energy $\left(\Delta E_{VaW}\right)$, and internal energy $\left(\Delta E_{inter}\right)$(bound interaction). $\Delta G_{Sol}$ is the total of both polar solvation (calculated using the born generalized model) and nonpolar solvation (calculated using the solvent accessible surface area). TΔS was calculated by normal mode analysis. However, this section was neglected because this study focused only on relative binding energies. The dielectric constant (ε) of the solvent was 78.5 and the surface tension constant of 0.03012 kJ mol^−1^ Å^2^ was used for the calculation of MM/GBSA. 

## 3. Results and Discussion

### 3.1. DENV-2 Sequences Collection

The first step in this study was to collect the NS5 region of RdRp of DENV-2 sequences from various countries. The collection of DENV sequences from diverse nations yielded 39 DENV-2 and 2 DENV-4 sequences for further study (Table 1). The DENV-4 sequences were served as outgroups. Some researchers suggested that RdRp can serve as the foundation for vaccine development [21,53,54,55]. 

### 3.2. Multiple Sequence Alignment

Multiple sequence alignment (MSA) was performed to determine the degree of similarity between each amino acids’ sequences. This technique is typically employed to examine the evolution of sequences from a common ancestor in order to identify sequence database commonalities [56]. The discrepancy in the alignment findings shows a mutation process in a sequence, whereas the gap suggests the presence of an insertion or deletion. Similarity between protein sequences can facilitate the identification of protein structure and function [57]. The MSA results (Supplementary File S1) reveal several variations that result in mismatches due to amino acid alterations. In addition, there is a gap characterized by amino acid sequence deletions. Mutations in the gene producing the NS5 protein generate the amino acids variations. A single mutation can be fatal to the virus. One finding revealed that the presence of a single mutation to alanine at NS5 position R888, which is a residue conserved in all Flavivirus, causes the virus to become completely non-viable [58]. This MSA was carried out to determine the conserved region of DENV-2, which will then be used to design peptide-based vaccine epitopes.

### 3.3. Phylogenetic Tree Analysis

**Figure 1:**
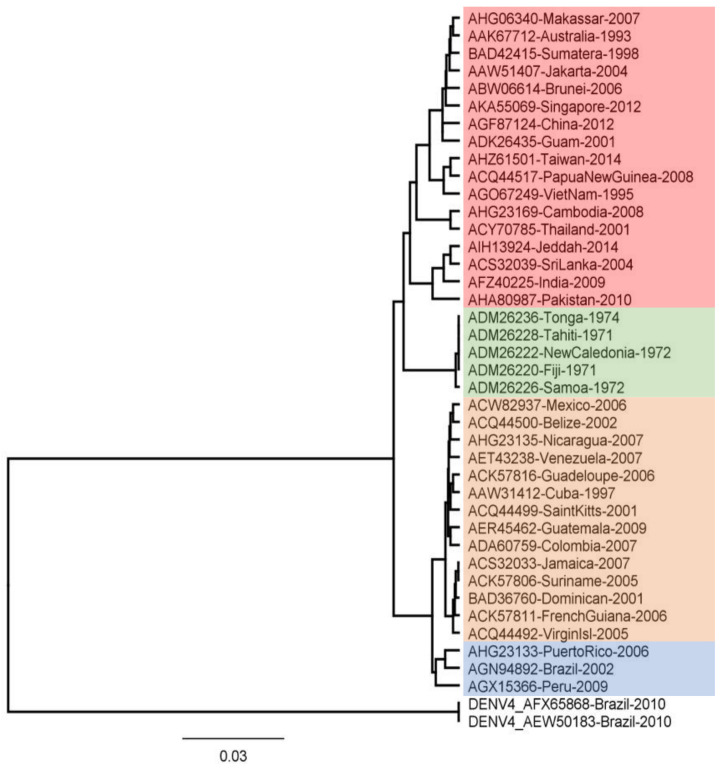
Evolutionary relationships of DENV-2 RNA-Dependent RNA Polymerase Protein. The evolutionary history was inferred using the UPGMA method. The evolutionary distances were computed using the Jukes–Cantor Method. Evolutionary analyses were conducted in Geneious. The DENV-4 sequences, which serve as outgroup, are from Brazil.

Phylogenetic tree analysis using molecular data, such as DNA or proteins, aims to construct precise relationships between genes or taxa. Figure 1 displays the findings of the reconstruction of the phylogenetic tree for the NS5 region. The tree consists of four clades for DENV-2 and one clade for DENV-4. The results of this reconstruction show that Indonesian specimens, particularly Sumatra and Jakarta, are in the same clade as Singapore. This indicates that these specimens are closely related. Sequences that are closely related can be identified by occupying neighboring branches in the tree [59]. The genotypes of the specimens are presented in Table 2. According to previous research, each DENV-2 genotype is neutralized differently by vaccine antibodies [60]. Therefore, it is assumed that the peptide-based vaccine epitope derived from the DENV-2 conserved region is a viable option.

### 3.4. B-Cell Epitope Analysis

**Figure 2:**
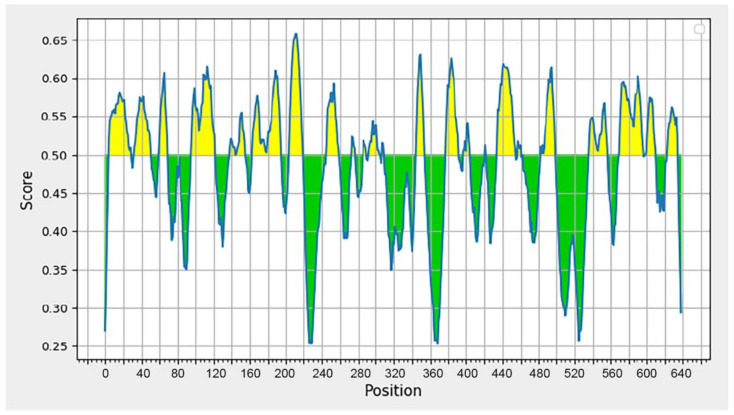
Graph displaying the results of B-cell epitope prediction using BepiPred Linear Epitope Prediction 2.0. The graph illustrates two prediction areas: the area with a yellow peak over the red line (threshold), indicating the area of the B-cell epitope, and the area with a green peak, which is not the prediction area for the B-cell epitope.

The BepiPred [61] was used for the analysis of B-cell epitopes because the application has a high prediction accuracy on the test data collection, and it has been demonstrated to perform significantly better than other methods. Antibodies in the immune system recognize a molecule by its B-cell epitopes [62]. The DENV-2 sequence from Sumatra (BAD42415) was used to predict B-cell epitopes. Figure 2 depicts the outcomes of the prediction. Residues with a score greater than 0.35 are displayed in yellow and are candidates for predicted epitopes [63]. 

There are several predicted peptides that seem able to bind antibodies, as shown in Table 3. A peptide vaccine consists of 8 to 30 amino acid residues and is considered adequate for eliciting the proper cellular and human immune response, while eliminating allergenic and/or reactogenic responses. These vaccines can also be used to protect against multiple strains or serological variants of a given pathogen [64]. Using this criteria, seven peptides with lengths ranging from 15 to 25 amino acids (shown in bold) were identified as prospective DENV-2 vaccine candidates. These peptides subsequently underwent predictive immunological testing.

### 3.5. Analysis of Epitope Immunological Properties

The selected candidate peptides for vaccines were next evaluated for immunological features, such as antigenicity, allergenicity, toxicity, and homology with human peptides. Vaccines must have antigenic potential, be non-allergenic, non-toxic, conserved, and not be identical to the human peptide/protein [65,66]. Table 4 displays the results of the predicted immunological properties of the vaccine candidates. Almost all of the peptides are antigenic, non-toxic, non-allergenic, and non-homologous to humans, and fit the criteria for epitope vaccine candidates except for GKVRKDIQQWEPSRGWNDWTQ. The peptide MGKREKKLGEFGKAKG, having an antigenicity score of 1.3969, was chosen as the epitope vaccine candidate. Additionally, this peptide is located in a conserved catalytic core domain of RdRp from the positive-sense single-stranded RNA [(+)ssRNA] viruses and closely related viruses. Its antigenicity score was the highest among the peptides studied, indicating that its ability as an antigen to bind to specific antibodies is highly strong.

### 3.6. Target Receptor Preparation

The receptor used is a dendritic cell-associated C-type lectin 2 (PDB ID: 3WBP), which is an innate receptor produced on antigen-presenting cells that recognizes glycosylated pathogens and self-glycoprotein [67]. The resolution value of this protein is 1.8 Å and it belongs to Homo sapiens. One of the criteria utilized in receptor selection is the resolution value, where a value less than 3 Å can impair the stability of the receptor during the molecular docking process [68]. A low-resolution score is indicative of a more stable receptor. Table 5 displays the outcomes of the validation of the quality of the protein structure using the Ramachandran Plot. Ramachandran plots are the “gold standard” for evaluating new crystal structures [34].

The results of the analysis show that 90.1% of non-glycine residues are in the most favored regions, 9.6% are in additional allowed regions, 0.1% are in generously allowed regions, and 0.1% are in disallowed regions. A good model, in addition to having very few residues in the disallowed regions, should also have a large number of residues in the most favored regions [35]. Thus, the outcomes of this analysis are, therefore, deemed satisfactory. Based on these factors, the protein structure of dendritic cells has a higher quality; thus, it can be stated that the quality of the produced structural model is also good.

### 3.7. Protein Modeling and Molecular Docking

The MGKREKKLGEFGKAKG peptide sequence was modeled using the PEP-FOLD webserver. In addition, the modeling result with the optimal conformation was chosen based on the minimum energy indicated by the optimized potential for efficient structure prediction (sOPEP). This sOPEP energy specifies the modeling structure of the peptide that is close to its original state so that it can interact with the target receptor and give high affinity [69]. The peptide was subsequently docked with protein of dendritic cells. The binding-free energy (BFE) value was used to assess the strength of the binding affinity, which indicates how strong a reversible bond can be formed between two or more molecules [70]. This is because the vaccine candidate must be able to interact well with the receptor in order to activate the immune response [71]. The interaction resulted in a BFE value of −474.4 kcal/mol. BFE is released whenever a ligand forms an association with a receptor, as this results in a reduction in the overall energy of the complex. The release of BFE also compensates for any transformation of the ligand from its minimum energy to its bound conformation with the receptor. This transformation can occur at any point during the process. As a result, the affinity of a ligand for a particular receptor increases in proportion to the amount of energy that is liberated as a result of the ligand’s binding to the receptor. The interaction results with the lowest BFE value are optimal. More negativity is better. With this BFE value, it is expected that the epitope vaccine candidate peptide has a very strong interaction with the receptor; hence, possessing a high immunogenic potential. 

**Figure 3:**
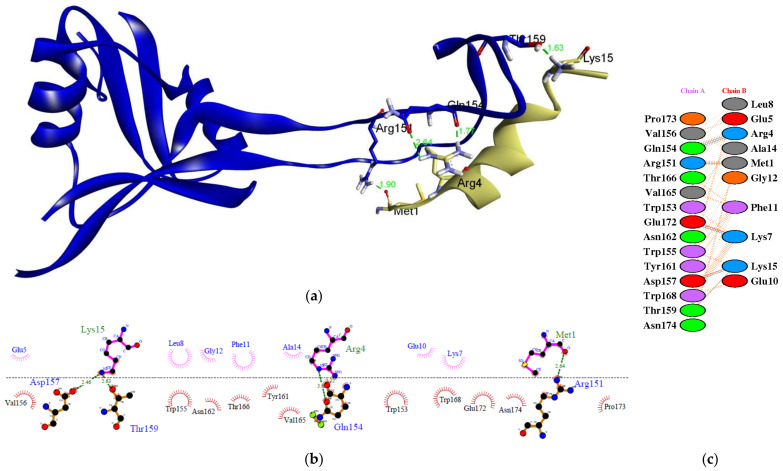
The molecular docking between the epitope MGKREKKLGEFGKAKG and paratope in the dendritic cells: (**a**) three-dimensional (3D) visualization showing that epitope binds in the pocket site of paratope; (**b**) two-dimensional (2D) visualization of the interaction of the complex using LigPlot+ V.2.2; (**c**) 2D visualization of the interaction generated from PDBSum webserver. The epitope is indicated in green color and the paratope is indicated in blue color.

Figure 3 depicts a molecular docking visualization. This visualization is required to ensure that the epitope can interact with the paratope at the dendritic cell receptor’s active site. According to the results of two-dimensional (2D) imaging, there are amino acid residues that establish hydrogen bonds (H-bonds) and hydrophobic interactions with the active site of the receptor. These amino acid residues form H-bonds with interaction distances ranging from 2.51 to 3.02 Å. There are seven H-bonds that bind to the active site region of the paratope at residues Arg151, Gln154, Asp157, Thr159, and Glu172. H-bonds contribute significantly to ligand–receptor interactions and contribute to the stability of the complex [72]. The angle at which the bond is formed is another important factor to consider. The strength of the hydrogen bond is directly proportional to the degree to which its geometry is accurate [73]. Additionally, the epitope interacts hydrophobically with the paratope at residues Trp153, Val156, Trp155, Tyr161, Asn162, Thr166, Val165, Trp168, Glu172, Pro173, and Asn174. There is also the formation of salt bridges between Lys15 and Asp157, as well as Lys7 and Glu 172. In many cases, the structural driving forces that increase the interaction stability are related to salt bridges [74]. The more bonds formed between the peptide and the active site of the receptor, the more stable the interaction, and the more negative the energy, the stronger the potential activity of the epitope vaccine.

### 3.8. Molecular Dynamics Simulations Study

Molecular dynamics simulations (MDS) are an advanced simulation used to examine the movement and interactions of molecules. On the basis of a general physics model that governs interatomic interactions, these simulations make predictions regarding the movement of each atom that makes up a protein or other molecular system over the course of time [75]. Moreover, MDS can be used to determine the conformational changes of the docked molecules [76]. The simulations are able to analyze the stability and the interaction mechanism formed between the complexes through the analysis of root-mean-square deviation (RMSD) and radius of gyration (Rg) during the simulation based on a predetermined time.

**Figure 4:**
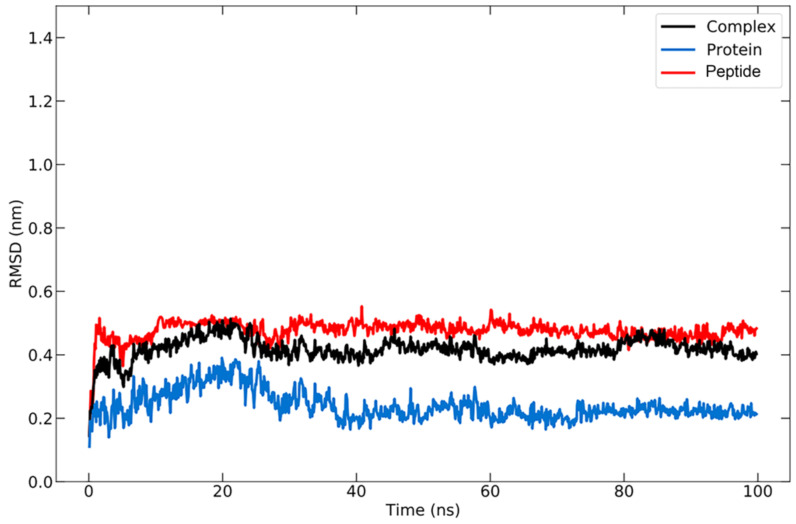
RMSD graph for protein backbone (in blue), peptide (in red), and the protein–peptide complex (in black) during 100 ns simulation.

RMSD analysis was carried out to compare the conformation of the structure at a certain time to the conformation of the initial structure. The RMSD for the protein–peptide complex was calculated based on the backbone atoms using the visual molecular dynamics (VMD) program, which resulted in an average value of 2.397 ± 0.449 Å (Figure 4). The RMSD graph for the protein backbone reveals that the structure undergoes an increase in RMSD for up to 20 ns, after which the structure remains stable with some fluctuations in the ~1 range, as is typical for globular proteins. The increase in RMSD indicates that the protein structure is beginning to unfold [77], at which point the peptide will search for the appropriate binding site or coordinates. A stable structure denotes that the conformation of the protein bound to the peptide has begun to be attained, allowing the protein to maintain its position. The average RMSD value for the peptide was 4.762 ± 0.311 Å. During the simulation, the RMSD of the peptide remained quite stable, indicating that it remained bound to the protein.

**Figure 5:**
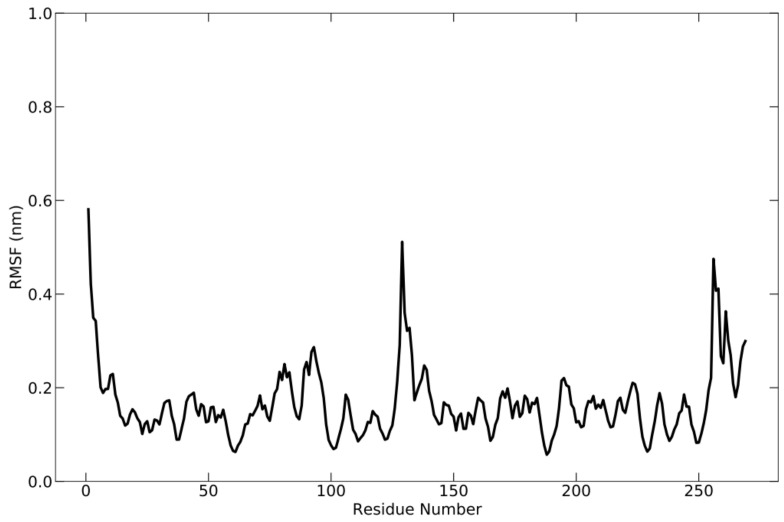
RMSF graph of the protein complex based on C-alpha atoms, which shows the fluctuation of each residue below 0.59 nm during simulation.

During the simulation, the flexibility of the complex was analyzed using root-mean-square fluctuation (RMSF) (Figure 5), which allowed for the acquisition of additional information regarding the fluctuating protein regions. RMSF is the square root of the average fluctuation between the positions of atoms and the reference structure. Protein flexibility is calculated for each amino acid residue to see how each residue fluctuates or moves during the simulation. During the simulation, the fluctuation occurred around 0.59 nm. This is demonstrated by the presence of multiple peaks on the graph.

**Figure 6:**
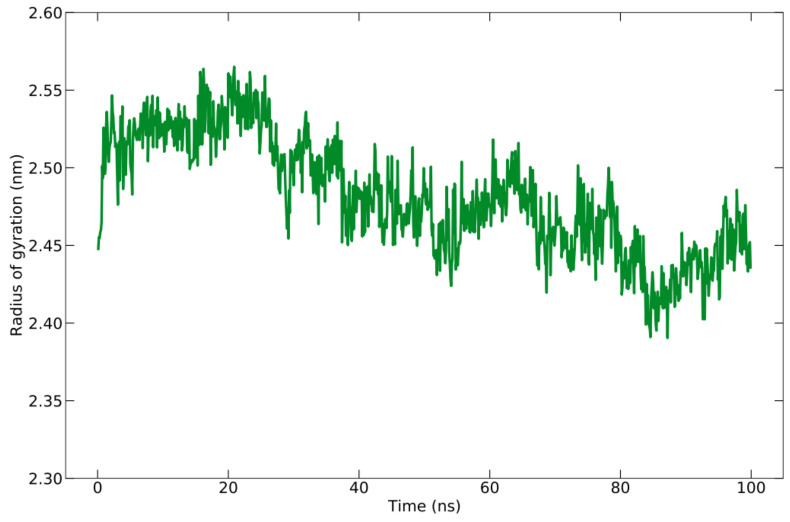
Rg graph during the simulation of 100 ns.

The compactness or density of protein molecules can be determined by analyzing Rg. The reduction in the number of fluctuations that occurred while running the simulation is evidence of the increased cohesiveness of the system [78]. The Rg for the protein–peptide complex is calculated based on the C-alpha atom with an average value of 24.815 ± 0.372 Å (Figure 6). There is a slight fluctuation with the Rg value of 1 Å during the simulation time, indicating a slight opening and closing of the N- and C-terminal domains.

**Figure 7:**
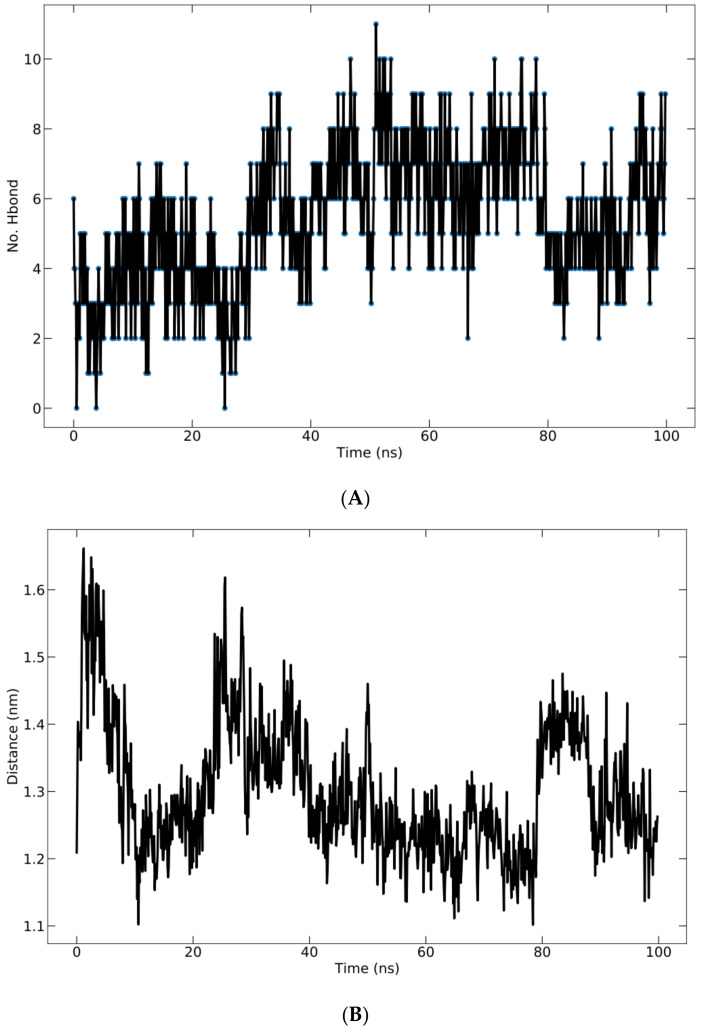
(**A**) Hydrogen bonding of protein–ligand complex; (**B**) average distance between peptide and protein during 100 ns simulation.

Hydrogen bonding in the complex is crucial for structure stability [33]. The total number of H-bonds formed between the protein and peptide during the 100 ns simulation is shown in Figure 7A. All ligands display a fluctuating number of H-bonds with the protein and maintain their bound state. The fluctuations indicated that the peptide altered the protein pocket’s conformation. The H-bonds occupancy was calculated as the conformational fraction of 1000 conformations of the protein–ligand complex in which a given residue participates in hydrogen bonding. The 1000 conformations of each complex are derived from the corresponding 100 ns molecular dynamics trajectory. Meanwhile, the average center of mass distance between the ligand and protein over 100 ns of simulation time is shown in Figure 7B. The mean distance is 13.00 ± 0.9766 Å. 

**Figure 8:**
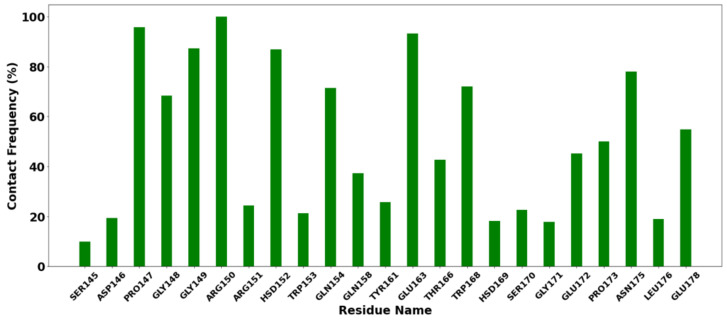
Histogram of contact frequency analysis with a cutoff of 4 Å.

Contact frequency (CF) analysis was performed to further evaluate the binding between the protein–ligands tested. Interactions between proteins and ligands are vitally important to almost every process that takes place in living organisms [79]. The analysis was performed using the contactFreq.tcl module in VMD with a cutoff of 4 Å. Residues with a higher percentage of CF (>90%) are Arg150, Pro147, HSD152 (prototropic tautomer of histidine), and Glu163 (Figure 8). This indicates that the interaction is within the cutoff, which is typical of van der Waals interactions.

**Figure 9:**
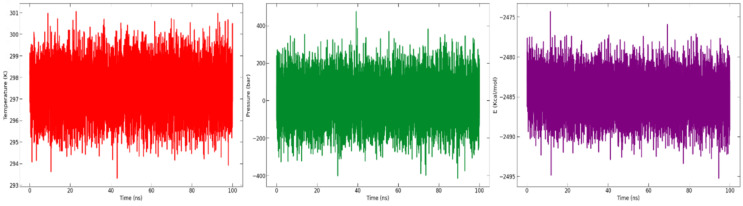
Graph of analysis of temperature (K) (in red), pressure (bar) (in green), and potential energy (kcal/mol) (in purple) throughout the 100 ns simulation time.

The potential energy, pressure, and temperature of the system over a 100 ns MD simulation, as obtained from the NAMD (nanoscale molecular dynamics) log file, is shown in Figure 9. The graph demonstrates that the potential energy, pressure, and temperature remained constant throughout the 100 ns simulation.

### 3.9. Binding-Free Energy

The molecular mechanics-generalized born surface area (MM-GBSA) method was chosen for complex rescoring because it is the fastest force field-based method that calculates binding-free energy compared to other computational free energy methods, such as free energy perturbation (FEP) or thermodynamic integration (TI) methods. Comparative studies also show that MM-GBSA outperform molecular mechanics Poisson–Boltzmann surface area (MM-PBSA) [80]. MM-GBSA calculations were conducted using the drug design software MolAICal, which combines artificial intelligence and classical programming [52]. The software provides a method for calculating MM-GBSA based on the output results of MDS performed by NAMD [42]. Table 6 displays the BFE results, which indicate that the ligand remained bound throughout the entire simulation time. 

## 4. Conclusions

Dengue fever is one of the most widespread and significant arboviral infections in the world. A licensed dengue vaccine is not yet effective against dengue serotypes in South East Asia, where dengue is endemic. Therefore, a peptide-based epitope vaccine is proposed as an alternative. According to the current findings based on the molecular docking study, the MGKREKKLGEFGKAKG epitope has the potential to be a candidate for a dengue vaccine with its BFE of −474.4 kcal/mol. This value indicates that the epitope and dendritic cells form a stable bond. The molecular dynamics simulation results showed that the protein–peptide complex only experienced a slight fluctuation at the beginning, and then remained in a stable state. According to this computational analysis, the suggested vaccine candidate will likely be structurally stable and predictably able to trigger an efficient immune response to thwart DENV-2 infections. However, experimental evaluation is still required to confirm the correct safety and immunogenicity profile of this vaccine in order to assess its potential.

## Figures and Tables

**Table 1:** The sequences of the NS5 region of DENV-2 RdRp from several countries accessed from NCBI.

Accession Number	Origin	Year of Sample Collection	Organism
ACQ44500	Belize	2002	*Homo sapiens*
AHG23135	Nicaragua	2007	*H. sapiens*
ABV02465	Mexico	2005	*H. sapiens*
AER45462	Guatemala	2009	*H. sapiens*
ADA60759	Colombia	2007	*H. sapiens*
AET43238	Venezuela	2007	*H. sapiens*
AGN94892	Brazil	2002	*H. sapiens*
AHG23133	Puerto Rico	2006	*H. sapiens*
AGX15366	Peru	2009	*H. sapiens*
AAW31412	Cuba	1997	*H. sapiens*
ACQ44499	Saint Kitts	2001	*H. sapiens*
ACK57816	Guadeloupe	2006	*H. sapiens*
BAD36760	Dominica	2001	*H. sapiens*
ACQ44492	Virgin Island	2005	*H. sapiens*
ACK57811	French Guiana	2006	*H. sapiens*
ACS32033	Jamaica	2007	*H. sapiens*
ACK57806	Suriname	2005	*H. sapiens*
AGO67249	Vietnam	1995	*H. sapiens*
AHG23169	Cambodia	2008	*H. sapiens*
ACY70785	Thailand	2001	*H. sapiens*
ACQ44517	Papua New Guinea	2008	*H. sapiens*
AHZ61501	Taiwan	2014	*H. sapiens*
AHA80987	Pakistan	2010	*H. sapiens*
AIH13924	Jeddah	2014	*H. sapiens*
ACS32039	Sri Lanka	2004	*H. sapiens*
AFZ40225	India	2009	*H. sapiens*
AGF87124	China	2012	*H. sapiens*
ADK26435	Guam	2001	*H. sapiens*
BAD42415	Sumatera-Indonesia	1998	*H. sapiens*
AHG06340	Makassar-Indonesia	2007	*H. sapiens*
AAW51407	Jakarta-Indonesia	2004	*H. sapiens*
AAK67712	Australia	1993	*H. sapiens*
AKA55069	Singapore	2012	*H. sapiens*
ABW06614	Brunei	2006	*H. sapiens*
ADM26236	Tonga	1974	*H. sapiens*
ADM26220	Fiji	1971	*H. sapiens*
ADM26228	Tahiti	1971	*H. sapiens*
ADM26222	New Caledonia	1972	*H. sapiens*
ADM26222	Samoa	1972	*H. sapiens*
AEW50183 (DENV-4)	Brazil	2010	*H. sapiens*
AFX65868 (DENV-4)	Brazil	2010	*H. sapiens*

**Table 2:** The genotypes of DENV-2 generated from their evolutionary relationship.

Cluster	Origin of Specimen
I (American Genotype)	Brazil, Puerto Rico, Peru
II (American Genotype)	▪ Dominican, Virgin Islands, Guadeloupe ▪ French Guiana, Jamaica, Suriname ▪ Belize, Mexico, Venezuela, Nicaragua ▪ Guatemala, Colombia, Cuba, Saint Kitts
III (Asian Genotype)	▪ Makassar, Australia, Jakarta, Sumatera, Brunei, Singapore, Guam, China ▪ Vietnam, Papua New Guinea, Taiwan ▪ Cambodia, Thailand ▪ Pakistan, India, Jeddah, Sri Lanka
IV (Pacific Islands)	Samoa, Tahiti, Tonga, Fiji, New Caledonia

**Table 3:** The predicted B-cell epitopes derived from the amino acid sequence of Sumatran specimen.

Start	End	Peptide	Length
5	29	**DVDLGSGTRNIGIESEIPNLDIIGK**	25
33	51	**KIKQEHETSWHYDQDHPYK**	19
60	69	ETKQTGSASS	10
97	122	TPFGQQRVFKEKVDTRTQEPKEGTKK	26
146	155	REEFTRKVRS	10
163	196	FTDENKWKSAREAVEDSGFWELVDKERNLHLEGK	34
205	220	**MGKREKKLGEFGKAKG**	16
246	259	HWFSRENSLSGVEG	14
293	306	TLEDLKNEEMVTNH	14
345	354	RRDQRGSGQV	10
378	392	**VFKSIQHLTVTEEIA**	15
398	405	ARVGRERL	8
435	455	**GKVRKDIQQWEPSRGWNDWTQ**	21
483	500	**ELIGRARISQGAGWSLRE**	18
537	557	**WVPTSRTTWSIHATHEWMTTE**	21
570	597	ENPWMEDKTPVESWEEIPYLGKREDQWC	28
601	611	IGLTSRATWAK	11
624	635	IGNEEYTDYMPS	12

**Table 4:** Predicted results of the immunological properties of the epitopes as vaccine candidates. The values are enclosed in brackets.

Epitope	Length	Antigenicity	Allergenicity	Toxicity	Homology to Human
DVDLGSGTRNIGIESEIPNLDIIGK	25	Antigen (1.0462)	Non- allergen (0.69)	Non-toxin (−0.78)	Non-Homolog
KIKQEHETSWHYDQDHPYK	19	Antigen (0.4439)	Non-allergen (0.64)	Non-toxin (−0.70)	Non-Homolog
MGKREKKLGEFGKAKG	16	Antigen (1.3969)	Non-allergen (0.59)	Non-toxin (−0.45)	Non-Homolog
GKVRKDIQQWEPSRGWNDWTQ	21	Non-antigen (−0.2845)	Non- allergen (0.64)	Non-toxin (−1.04)	Non-Homolog
VFKSIQHLTVTEEIA	15	Antigen (0.3651)	Non-allergen (0.64)	Non-toxin (−1.17)	Non-Homolog
ELIGRARISQGAGWSLRE	18	Antigen (0.6645)	Non-allergen (0.59)	Non-toxin (−1.43)	Non-Homolog
WVPTSRTTWSIHATHEWMTTE	21	Antigen (0.8736)	Non-allergen (0.67)	Non-toxin (−1.50)	Non-Homolog

**Table 5:** The results of the validation of the quality of the 3WBP structure using a PROCHECK program that relies on the Ramachandran Plot.

Ramachandran Criteria	Percentage
Most Favored Regions	90.1%
Additional allowed regions	9.6%
Generously allowed regions	0.1%
Disallowed regions	0.1%

**Table 6:** The binding-free energy (kcal/mol) of the complex calculated using MM/GBSA method.

ΔG	$\Delta E_{\left(internal\right)}$	$\Delta E_{\left(electrostat\right)}+\Delta G_{\left(sol\right)}$	$\Delta E_{\left(VDW\right)}$
−35.784 ± 0.2933	0	3.0796	−38.8636

## Data Availability

Not applicable.

## Supplementary Materials

The following supporting information can be downloaded at: https://www.mdpi.com/article/10.3390/vaccines10101734/s1, Supplementary File S1—Multiple Sequence Alignment.

## References

[1] Cox J., Mota J., Sukupolvi-Petty S., Diamond M.S., Rico-Hesse R. (2012). Mosquito Bite Delivery of Dengue Virus Enhances Immunogenicity and Pathogenesis in Humanized Mice. J. Virol..

[2] Bhatt S., Gething P.W., Brady O.J., Messina J.P., Farlow A.W., Moyes C.L., Drake J.M., Brownstein J.S., Hoen A.G., Sankoh O. (2013). The Global Distribution and Burden of Dengue. Nature.

[3] Rothman A.L., Ennis F.A. (2016). Dengue Vaccine: The Need, the Challenges, and Progress. J. Infect. Dis..

[4] Olivera-Botello G., Coudeville L., Fanouillere K., Guy B., Chambonneau L., Noriega F., Jackson N. (2016). Tetravalent Dengue Vaccine Reduces Symptomatic and Asymptomatic Dengue Virus Infections in Healthy Children and Adolescents Aged 2-16 Years in Asia and Latin America. J. Infect. Dis..

[5] Marintcheva B. (2018). Chapter 8—Viruses as tools for vaccine, development. Harnessing the Power of Viruses.

[6] Tam J.P. (1988). Synthetic Peptide Vaccine Design: Synthesis and Properties of a High-Density Multiple Antigenic Peptide System. Proc. Natl. Acad. Sci. USA.

[7] Dillon P.M., Slingluff C.L., Marshall J.L. (2017). Peptide Vaccine: Overview. Cancer Therapeutic Targets.

[8] Murugesan A., Manoharan M. (2020). Dengue Virus. Emerging and Reemerging Viral Pathogens.

[9] Gubler D.J. (1998). Dengue and Dengue Hemorrhagic Fever. Clin. Microbiol. Rev..

[10] Wang W.-H., Urbina A.N., Chang M.R., Assavalapsakul W., Lu P.-L., Chen Y.-H., Wang S.-F. (2020). Dengue Hemorrhagic Fever—A Systemic Literature Review of Current Perspectives on Pathogenesis, Prevention and Control. J. Microbiol. Immunol. Infect..

[11] Norshidah H., Vignesh R., Lai N.S. (2021). Updates on Dengue Vaccine and Antiviral: Where Are We Heading?. Molecules.

[12] Khan N.U., Danish L., Khan H.U., Shah M., Ismail M., Ali I., Petruzziello A., Sabatino R., Guzzo A., Botti G. (2020). Prevalence of Dengue Virus Serotypes in the 2017 Outbreak in Peshawar, KP, Pakistan. J. Clin. Lab. Anal..

[13] Fried J.R., Gibbons R.V., Kalayanarooj S., Thomas S.J., Srikiatkhachorn A., Yoon I.-K., Jarman R.G., Green S., Rothman A.L., Cummings D.A.T. (2010). Serotype-Specific Differences in the Rrsk of Dengue Hemorrhagic Fever: An Analysis of Data Collected in Bangkok, Thailand from 1994 to 2006. PLoS Negl. Trop. Dis..

[14] Vicente C.R., Herbinger K.-H., Fröschl G., Malta Romano C., de Souza Areias Cabidelle A., Cerutti Junior C. (2016). Serotype Influences on Dengue Severity: A Cross-Sectional Study on 485 Confirmed Dengue Cases in Vitória, Brazil. BMC Infect. Dis..

[15] Martina B.E.E., Koraka P., Osterhaus A.D.M.E. (2009). Dengue Virus Pathogenesis: An Integrated View. Clin. Microbiol. Rev..

[16] Norazharuddin H., Lai N.S. (2018). Roles and Prospects of Dengue Virus Non-Structural Proteins as Antiviral Targets: An Easy Digest. Malays. J. Med. Sci..

[17] Davidson A.D. (2009). Chapter 2 New Insights into Flavivirus Nonstructural Protein 5. Advances in Virus Research.

[18] Gebhard L.G., Filomatori C.V., Gamarnik A.V. (2011). Functional RNA Elements in the Dengue Virus Genome. Viruses.

[19] Obi J.O., Gutiérrez-Barbosa H., Chua J.V., Deredge D.J. (2021). Current Trends and Limitations in Dengue Antiviral Research. Trop. Med. Infect. Dis..

[20] Shimizu H., Saito A., Mikuni J., Nakayama E.E., Koyama H., Honma T., Shirouzu M., Sekine S.-I., Shioda T. (2019). Discovery of a Small Molecule Inhibitor Targeting Dengue Virus NS5 RNA-Dependent RNA Polymerase. PLoS Negl. Trop. Dis..

[21] Alves R.P.D.S., Pereira L.R., Fabris D.L.N., Salvador F.S., Santos R.A., Zanotto P.M.d.A., Romano C.M., Amorim J.H., Ferreira L.C.d.S. (2016). Production of a Recombinant Dengue Virus 2 NS5 Protein and Potential Use as a Vaccine Antigen. Clin. Vaccine Immunol..

[22] Fadaka A.O., Sibuyi N.R.S., Martin D.R., Goboza M., Klein A., Madiehe A.M., Meyer M. (2021). Immunoinformatics Design of a Novel Epitope-Based Vaccine Candidate against Dengue Virus. Sci. Rep..

[23] Sami S.A., Marma K.K.S., Mahmud S., Khan M.A.N., Albogami S., El-Shehawi A.M., Rakib A., Chakraborty A., Mohiuddin M., Dhama K. (2021). Designing of a Multi-Epitope Vaccine against the Structural Proteins of Marburg Virus Exploiting the Immunoinformatics Approach. ACS Omega.

[24] Uno N., Ross T.M. (2018). Dengue Virus and the Host Innate Immune Response. Emerg. Microbes Infect..

[25] Pati R., Shevtsov M., Sonawane A. (2018). Nanoparticle Vaccines against Infectious Diseases. Front. Immunol..

[26] Zaneti A.B., Yamamoto M.M., Sulczewski F.B., Almeida B.d.S., Souza H.F.S., Ferreira N.S., Maeda D.L.N.F., Sales N.S., Rosa D.S., Ferreira L.C.d.S. (2019). Dendritic Cell Targeting Using a DNA Vaccine Induces Specific Antibodies and CD4(+) T Cells to the Dengue Virus Envelope Protein Domain III. Front. Immunol..

[27] Cohn L., Delamarre L. (2014). Dendritic Cell-Targeted Vaccines. Front. Immunol..

[28] Jonny J., Putranto T.A., Sitepu E.C., Irfon R. (2022). Dendritic Cell Vaccine as a Potential Strategy to End the COVID-19 Pandemic. Why Should It Be Ex Vivo?. Expert Rev. Vaccines.

[29] Liu K., Bradshaw R.A., Stahl P.D. (2016). Dendritic Cells. Encyclopedia of Cell Biology.

[30] Kastenmüller W., Kastenmüller K., Kurts C., Seder R.A. (2014). Dendritic Cell-Targeted Vaccines—Hope or Hype?. Nat. Rev. Immunol..

[31] Tarassoff C.P., Arlen P.M., Gulley J.L. (2006). Therapeutic Vaccines for Prostate Cancer. Oncologist.

[32] Rahman M.M., Puspo J.A., Adib A.A., Hossain M.E., Alam M.M., Sultana S., Islam A., Klena J.D., Montgomery J.M., Satter S.M. (2022). An Immunoinformatics Prediction of Novel Multi-Epitope Vaccines Candidate against Surface Antigens of Nipah Virus. Int. J. Pept. Res. Ther..

[33] Tallei T.E., Tumilaar S.G., Niode N.J., Fatimawali F., Kepel B.J., Idroes R., Effendi Y., Sakib S.A., Emran T. (2020). Bin Potential of Plant Bioactive Compounds as SARS-CoV-2 Main Protease (Mpro) and Spike (S) Glycoprotein Inhibitors: A Molecular Docking Study. Scientifica.

[34] Laskowski R.A., MacArthur M.W., Moss D.S., Thornton J.M. (1993). PROCHECK: A Program to Check the Stereochemical Quality of Protein Structures. J. Appl. Crystallogr..

[35] Kleywegt G.J., Jones T.A. (1996). Phi/Psi-Chology: Ramachandran Revisited. Structure.

[36] Kozakov D., Hall D.R., Xia B., Porter K.A., Padhorny D., Yueh C., Beglov D., Vajda S. (2017). The ClusPro Web Server for Protein-Protein Docking. Nat. Protoc..

[37] Laskowski R.A., Jabłońska J., Pravda L., Vařeková R.S., Thornton J.M. (2018). PDBsum: Structural Summaries of PDB Entries. Protein Sci..

[38] Brooks B.R., Brooks C.L., Mackerell A.D., Nilsson L., Petrella R.J., Roux B., Won Y., Archontis G., Bartels C., Boresch S. (2009). CHARMM: The Biomolecular Simulation Program. J. Comput. Chem..

[39] Jo S., Kim T., Iyer V.G., Im W. (2008). CHARMM-GUI: A Web-Based Graphical User Interface for CHARMM. J. Comput. Chem..

[40] Lee J., Cheng X., Swails J.M., Yeom M.S., Eastman P.K., Lemkul J.A., Wei S., Buckner J., Jeong J.C., Qi Y. (2016). CHARMM-GUI Input Generator for NAMD, GROMACS, AMBER, OpenMM, and CHARMM/OpenMM Simulations Using the CHARMM36 Additive Force Field. J. Chem. Theory Comput..

[41] Best R.B., Zhu X., Shim J., Lopes P.E.M., Mittal J., Feig M., Mackerell A.D.J. (2012). Optimization of the Additive CHARMM All-Atom Protein Force Field Targeting Improved Sampling of the Backbone φ, ψ and Side-Chain χ(1) and χ(2) Dihedral Angles. J. Chem. Theory Comput..

[42] Phillips J.C., Braun R., Wang W., Gumbart J., Tajkhorshid E., Villa E., Chipot C., Skeel R.D., Kalé L., Schulten K. (2005). Scalable Molecular Dynamics with NAMD. J. Comput. Chem..

[43] Price D.J., Brooks C.L. (2004). A Modified TIP3P Water Potential for Simulation with Ewald Summation. J. Chem. Phys..

[44] Nosé S. (1984). A Molecular Dynamics Method for Simulations in the Canonical Ensemble. Mol. Phys..

[45] Nosé S., Klein M.L. (1983). Constant Pressure Molecular Dynamics for Molecular Systems. Mol. Phys..

[46] Grest G.S., Kremer K. (1986). Molecular Dynamics Simulation for Polymers in the Presence of a Heat Bath. Phys. Rev. A Gen. Phys..

[47] Darden T., York D., Pedersen L. (1993). Particle Mesh Ewald: An N⋅log(N) Method for Ewald Sums in Large Systems. J. Chem. Phys..

[48] Essmann U., Perera L., Berkowitz M.L., Darden T., Lee H., Pedersen L.G. (1995). A Smooth Particle Mesh Ewald Method. J. Chem. Phys..

[49] Ryckaert J.-P., Ciccotti G., Berendsen H.J.C. (1977). Numerical Integration of the Cartesian Equations of Motion of a System with Constraints: Molecular Dynamics of n-Alkanes. J. Comput. Phys..

[50] Genheden S., Ryde U. (2012). Comparison of End-Point Continuum-Solvation Methods for the Calculation of Protein-Ligand Binding Free Energies. Proteins.

[51] Wang E., Sun H., Wang J., Wang Z., Liu H., Zhang J.Z.H., Hou T. (2019). End-Point Binding Free Energy Calculation with MM/PBSA and MM/GBSA: Strategies and Applications in Drug Design. Chem. Rev..

[52] Bai Q., Tan S., Xu T., Liu H., Huang J., Yao X. (2020). MolAICal: A Soft Tool for 3D Drug Design of Protein Targets by Artificial Intelligence and Classical Algorithm. Brief. Bioinform..

[53] Muhammed Y., Yusuf Nadabo A., Pius M., Sani B., Usman J., Anka Garba N., Mohammed Sani J., Opeyemi Olayanju B., Zeal Bala S., Garba Abdullahi M. (2021). SARS-CoV-2 Spike Protein and RNA Dependent RNA Polymerase as Targets for Drug and Vaccine Development: A Review. Biosaf. Health.

[54] Liu X., Yang X., Lee C.A., Moustafa I.M., Smidansky E.D., Lum D., Arnold J.J., Cameron C.E., Boehr D.D. (2013). Vaccine-Derived Mutation in Motif D of Poliovirus RNA-Dependent RNA Polymerase Lowers Nucleotide Incorporation Fidelity. J. Biol. Chem..

[55] Oany A.R., Sharmin T., Chowdhury A.S., Jyoti T.P., Hasan M.A. (2015). Highly Conserved Regions in Ebola Virus RNA Dependent RNA Polymerase May Be Act as a Universal Novel Peptide Vaccine Target: A Computational Approach. Silico Pharmacol..

[56] Yu Y., Santat L.A., Choi S., Arora D.K., Berka R.M., Singh G.B. (2006). 6-Bioinformatics Packages for Sequence Analysis. Applied Mycology and Biotechnology.

[57] Louie B., Higdon R., Kolker E. (2009). A Statistical Model of Protein Sequence Similarity and Function Similarity Reveals Overly-Specific Function Predictions. PLoS ONE.

[58] Tay M.Y.F., Smith K., Ng I.H.W., Chan K.W.K., Zhao Y., Ooi E.E., Lescar J., Luo D., Jans D.A., Forwood J.K. (2016). The C-Terminal 18 Amino Acid Region of Dengue Virus NS5 Regulates Its Subcellular Localization and Contains a Conserved Arginine Residue Essential for Infectious Virus Production. PLoS Pathog..

[59] Tallei T.E., Kolondam B.J. (2015). DNA Barcoding of Sangihe Nutmeg (Myristica Fragrans) Using MatK Gene. HAYATI J. Biosci..

[60] Martinez D.R., Yount B., Nivarthi U., Munt J.E., Delacruz M.J., Whitehead S.S., Durbin A.P., de Silva A.M., Baric R.S. (2020). Antigenic Variation of the Dengue Virus 2 Genotypes Impacts the Neutralization Activity of Human Antibodies in Vaccinees. Cell Rep..

[61] Jespersen M.C., Peters B., Nielsen M., Marcatili P. (2017). BepiPred-2.0: Improving Sequence-Based B-Cell Epitope Prediction Using Conformational Epitopes. Nucleic Acids Res..

[62] Larsen J.E.P., Lund O., Nielsen M. (2006). Improved Method for Predicting Linear B-Cell Epitopes. Immunome Res..

[63] Javadi Mamaghani A., Arab-Mazar Z., Heidarzadeh S., Ranjbar M.M., Molazadeh S., Rashidi S., Niazpour F., Naghi Vishteh M., Bashiri H., Bozorgomid A. (2021). In-Silico Design of a Multi-Epitope for Developing Sero-Diagnosis Detection of SARS-CoV-2 Using Spike Glycoprotein and Nucleocapsid Antigens. Netw. Model. Anal. Health Inform. Bioinform..

[64] Li W., Joshi M.D., Singhania S., Ramsey K.H., Murthy A.K. (2014). Peptide Caccine: Progress and Challenges. Vaccines.

[65] Sarkar B., Ullah M.A., Araf Y., Islam N.N., Zohora U.S. (2021). Immunoinformatics-Guided Designing and in Silico Analysis of Epitope-Based Polyvalent Vaccines against Multiple Strains of Human Coronavirus (HCoV). Expert Rev. Vaccines.

[66] Yang Z., Bogdan P., Nazarian S. (2021). An in Silico Deep Learning Approach to Multi-Epitope Vaccine Design: A SARS-CoV-2 Case Study. Sci. Rep..

[67] Bloem K., Vuist I.M., van der Plas A.-J., Knippels L.M.J., Garssen J., García-Vallejo J.J., van Vliet S.J., van Kooyk Y. (2013). Ligand Binding and Signaling of Dendritic Cell Immunoreceptor (DCIR) Is Modulated by the Glycosylation of the Carbohydrate Recognition Domain. PLoS ONE.

[68] Marcou G., Rognan D. (2007). Optimizing Fragment and Scaffold Docking by Use of Molecular Interaction Fingerprints. J. Chem. Inf. Model..

[69] Thévenet P., Shen Y., Maupetit J., Guyon F., Derreumaux P., Tufféry P. (2012). PEP-FOLD: An Updated de Novo Structure Prediction Server for Both Linear and Disulfide Bonded Cyclic Peptides. Nucleic Acids Res..

[70] Kastritis P.L., Bonvin A.M.J.J. (2013). On the Binding Affinity of Macromolecular Interactions: Daring to Ask Why Proteins Interact. J. R. Soc. Interface.

[71] Sadarangani M., Marchant A., Kollmann T.R. (2021). Immunological Mechanisms of Vaccine-Induced Protection against COVID-19 in Humans. Nat. Rev. Immunol..

[72] Abelian A., Dybek M., Wallach J., Gaye B., Adejare A., Adejare A.B. (2021). Chapter 6—Pharmaceutical Chemistry. Remington: The Science and Practice of Pharmacy.

[73] McRee D.E., McREE D.E. (1999). 3-Computational Techniques. Practical Protein Crystallography.

[74] Pylaeva S., Brehm M., Sebastiani D. (2018). Salt Bridge in Aqueous Solution: Strong Structural Motifs but Weak Enthalpic Effect. Sci. Rep..

[75] Karplus M., McCammon J.A. (2002). Molecular Dynamics Simulations of Biomolecules. Nat. Struct. Biol..

[76] Radwan A., Mahrous G.M. (2020). Docking Studies and Molecular Dynamics Simulations of the Binding Characteristics of Waldiomycin and Its Methyl Ester Analog to Staphylococcus Aureus Histidine Kinase. PLoS ONE.

[77] Zhou R., Eleftheriou M., Hon C.-C., Germain R.S., Royyuru A.K., Berne B.J. (2008). Massively Parallel Molecular Dynamics Simulations of Lysozyme Unfolding. IBM J. Res. Dev..

[78] Lobanov M.Y., Bogatyreva N.S., Galzitskaya O.V. (2008). Radius of Gyration as an Indicator of Protein Structure Compactness. Mol. Biol..

[79] Becker W., Bhattiprolu K.C., Gubensäk N., Zangger K. (2018). Investigating Protein-Ligand Interactions by Solution Nuclear Magnetic Resonance Spectroscopy. Chemphyschem.

[80] Hou T., Wang J., Li Y., Wang W. (2011). Assessing the Performance of the MM/PBSA and MM/GBSA Methods. 1. The Accuracy of Binding Free Energy Calculations Based on Molecular Dynamics Simulations. J. Chem. Inf. Model..

