# Supplementary material for: Designing an Epitope-Based Peptide Vaccine Derived from RNA-Dependent RNA Polymerase (RdRp) against Dengue Virus Serotype 2
Source: Vaccines (Basel). 2022 Oct 17;10(10):1734. doi: 10.3390/vaccines10101734 (PMC9611443; doi:10.3390/vaccines10101734)
Supplement: Supplementary file 1 [file vaccines-1921717-supplementary.pdf › vaccines-1921717-supplementary.pdf]

Clustal Omega

Results for job clustalo-I20220715-121525-0370-87297613-p1m

Alignments | Result Summary | Guide Tree | Phylogenetic Tree | Results Viewers | Submission Details

Download Alignment File | Hide Colors

```
CLUSTAL O(1.2.4) multiple sequence alignment


DENV4_AEW50183-Brazil-2010      TYEKDVDLGAGTRSVSTETEKPDMTIIGRRLQRLQEEHKETWHYDQENPYRTWAYHGSYE	60
DENV4_AFX65868-Brazil-2010      TYEKDVDLGAGTRSVSTETEKPDMTIIGRRLQRLQEEHKETWHYDQENPYRTWAYHGSYE	60
AHA80987-Pakistan-2010          TYEPDVDLGSGTRNIGIESETPNLDIIGKRIEKIKQEHETSWHYDQDHPYKTWAYHGSYE	60
AFZ40225-India-2009             TYEPDVDLGSGTRNIGIESETPNLDIIGKRIEKIKQEHETSWHYDQDHPYKTWAYHGSYE	60
AIH13924-Jeddah-2014            TYEPDVDLGSGTRNIGIESETPNLDIIGKRIEKIKQEHETSWHYDQDHPYKTWAYHGSYE	60
ACS32039-SriLanka-2004          TYEPDVDLGSGTRNIGIESETPNLDIIGKRIEKIKQEHETSWHYDQDHPYKTWAYHGSYE	60
ADM26228-Tahiti-1971            TYEPDVDLGSGTRNIGIESETPNLDIIGKRIEKIKQEHETSWHYDQDHPYKTWAYHGSYE	60
ADM26226-Samoa-1972             TYEPDVDLGSGTRNIGIESETPNLDIIGKRIEKIKQEHETSWHYDQDHPYKTWAYHGSYE	60
ADM26236-Tonga-1974             TYEPDVDLGSGTRNIGIESETPNLDIIGKRIEKIKQEHETSWHYDQDHPYKTWAYHGSYE	60
ADM26220-Fiji-1971              TYEPDVDLGSGTRNIGIESETPNLDIIGKRIEKIKQEHETSWHYDQDHPYKTWAYHGSYE	60
ADM26222-NewCaledonia-1972      TYEPDVDLGSGTRNIGIESETPNLDIIGKRIEKIKQEHETSWHYDQDHPYKTWAYHGSYE	60
ACQ44499-SaintKitts-2001        TYEPDVDLGSGTRNIGIESETPNLDIIGKRIEKIKQEHETSWHYDQDHPYKTWAYHGSYE	60
AER45462-Guatemala-2009         TYEPDVDLGSGTRNIGIESEVPNLDIIGKRIEKIKQEHETSWHYDQDHPYKTWAYHGSYE	60
ADA60759-Colombia-2007          TYEPDVDLGSGTRNIGIESEIPNLDIIGKRIEKIKQEHETSWHYDQDHPYKTWAYHGSYE	60
ACK57816-Guadeloupe-2006        TYEPDVDLGSGTRNIGIESEIPNLDIIGKRIEKIKQEHETSWHYDQDHPYKTWAYHGSYE	60
BAD36760-Dominican-2001         TYEPDVDLGSGTRNIGIESEIPNLDIIGKRIEKIKQEHETSWHYDQDHPYKTWAYHGSYE	60
ACQ44492-VirginIsl-2005         TYEPDVDLGSGTRNIGIESEIPNLDIIGKRIEKIKQEHETSWHYDQDHPYKTWAYHGSYE	60
ACK57811-FrenchGuiana-2006      TYEPDVDLGSGTRNIGIESEIPNLDIIGKRIEKIKQEHETSWHYDQDHPYKTWAYHGSYE	60
ACS32033-Jamaica-2007           TYEPDVDLGSGTRNIGIESEIPNLDIIGKRIEKIKQEHETSWHYDQDHPYKTWAYHGSYE	60
ACK57806-Suriname-2005          TYEPDVDLGSGTRNIGIESEIPNLDIIGKRIEKIKQEHETSWHYDQDHPYKTWAYHGSYE	60
AAW31412-Cuba-1997              TYEPDVDLGSGTRNIGIESEIPNLDIIGKRIEKIKQEHETSWHYDQDHPYKTWAYHGSYE	60
AET43238-Venezuela-2007         TYEPDVDLGSGTRNIGIESEIPNLDIIGKRIEKIKQEHETSWHYDQDHPYKTWAYHGSYE	60
ACQ44500-Belize-2002            TYEPDVDLGSGTRNIGIESEIPNLDIIGKRIEKIKQEHETSWHYDQDHPYKTWAYHGSYE	60
ACW82937-Mexico-2006            TYEPDVDLGSGTRNIGIESEIPNLDIIGKRIEKIKREHETSWHYDQDHPYKTWAYHGSYE	60
AHG23135-Nicaragua-2007         TYEPDVDLGSGTRNIGIESEIPNLDIIGKRIEKIKQEHEISWHYDQDHPYKTWAYHGSYE	60
AGX15366-Peru-2009              TYEPDVDLGSGTRNIGIESETPNLDIIGKRIEKIKQEHETSWHYDQDHPYKTWAYHGSYE	60
AGN94892-Brazil-2002            TYEPDVDLGSGTRNIGIESEIPNLDIIGKRIEKIKQEHETSWHYDQDHPYKTWAYHGSYE	60
AHG23133-PuertoRico-2006        TYEPDVDLGSGTRNIGIENEIPNLDIIGKRIEKIKQEHETSWHYDQDHPYKTWAYHGSYE	60
ADK26435-Guam-2001              TYEPDVDLGSGTRNIGIESETPNLDIIGKRIEKIKQEHETSWHYDQDHPYKTWAYHGSYE	60
AGF87124-China-2012             TYEPDVDLGSGTRNIGIESETPNLDIIGKRIEKIKQEHETSWHYDQDHPYKTWAYHGSYE	60
ABW06614-Brunei-2006            TYEPDVDLGSGTRNIGIESETPDLDIIGKRIEKIKQEHETSWHYDQDHPYKTWAYHGSYE	60
AAW51407-Jakarta-2004           TYEPDVDLGSGTRNIGIESETPNLDIIGKRIEKIKQEHETSWHYDQDHPYKTWAYHGSYE	60
AKA55069-Singapore-2012         TYEPDVDLGSGTRNIGIESETPNLDIIGKRIEKIKQEHETSWHYDQDHPYKTWAYHGSYE	60
BAD42415-Sumatera-1998          TYEPDVDLGSGTRNIGIESEIPNLDIIGKRIEKIKQEHETSWHYDQDHPYKTWAYHGSYE	60
AHG06340-Makassar-2007          TYEPDVDLGSGTRNIGIESETPNLDIIGKRIEKIKQEHETSWHYDQDHPYKTWAYHGSYE	60
AAK67712-Australia-1993         TYEPDVDLGSGTRNIGIESETPNLDIIGKRIEKIKQEHETSWHYDQDHPYKTWAYHGSYE	60
AHG23169-Cambodia-2008          TYEPDVDLGSGTRNIGIESETPNLDIIGKRIEKIKEEHETSWHYDQDHPYKTWAYHGSYE	60
ACY70785-Thailand-2001          TYEPDVDLGSGTRNIGIESEIPNLDIIGKRIEKIKQEHETSWHYDQDHPYKTWAYHGSYE	60
AGO67249-VietNam-1995           TYEPDVDLGSGTRNIGIESEIPNLDIIGKRIEKIKQEHETSWHYDQDHPYKTWAYHGSYE	60
ACQ44517-PapuaNewGuinea-2008    TYEPDVDLGSGTRNIGIESEIPNLDIIGKRIEKIKQEHETSWHYDQDHPYKTWAYHGSYE	60
AHZ61501-Taiwan-2014            TYEPDVDLGSGTRNIGIESEVPNLDIIGKRIEKIKQEHETSWHYDQDHPYKTWAYHGSYE	60
                                *** *****:***.:. *.* *:: ***:*:::::.**: :*****::**:*********

DENV4_AEW50183-Brazil-2010      APSTGSASSMVNGVVKLLTKPWDVIPMVTQLAMTDTTPFGQQRVFKEKVDTRTPQPKPGT	120
DENV4_AFX65868-Brazil-2010      APSTGSASSMVNGVVKLLTKPWDVIPMVTQLAMTDTTPFGQQRVFKEKVDTRTPQPKPGT	120
AHA80987-Pakistan-2010          TKQTGSASSVVNGVVRLLTKPWDVIPMVTQMAMTDTTPFGQQRVFKEKVDTRTQEPKEGT	120
AFZ40225-India-2009             TKQTGSASSMVNGVVRLLTKPWDVIPMVTQMAMTDTTPFGQQRVFKEKVDTRTQEPKEGT	120
AIH13924-Jeddah-2014            TKQTGSASSMVNGVVRLLTKPWDVIPMVTQMAMTDTTPFGQQRVFKEKVDTRTQEPKEGT	120
ACS32039-SriLanka-2004          TKQTGSASSMVNGVVRLLTKPWDVIPMVTQMAMTDTTPFGQQRVFKEKVDTRTQEPKEGT	120
ADM26228-Tahiti-1971            TKQTGSASSMVNGVVRLLTKPWDVVPXVTQMAMTDTTPFGQQRVFKEKVDTRTQEPKEGT	120
ADM26226-Samoa-1972             TKQTGSASSMVNGVVRLLTKPWDVVPMVTQMAMTDTTPFGQQRVFKEKVDTRTQEPKEGT	120
ADM26236-Tonga-1974             TKQTGSASSMVNGVVRLLTKPWDVVPMVTQMAMTDTTPFGQQRVFKEKVDTRTQEPKEGT	120
ADM26220-Fiji-1971              TKQTGSASSMVNGVVRLLTKPWDVVPMVTQMAMTDTTPFGQQRVFKEKVDTRTQEPKEGT	120
ADM26222-NewCaledonia-1972      TKQTGSASSMVNGVVRLLTKPWDVVPMVTQMAMTDTTPFGQQRVFKEKVDTRTQEPKEGT	120
ACQ44499-SaintKitts-2001        TKQTGSASSMVNGVVRLLTKPWDVVPMVTQMAMTDTTPFGQQRVFKEKVDTRTQEPKEGT	120
AER45462-Guatemala-2009         TKQTGSASSMVNGVVRLLTKPWDVVPMVTQMAMTDTTPFGQQRVFKEKVDTRTQEPKEGT	120
ADA60759-Colombia-2007          TKQTGSASSMVNGVVRLLTKPWDVVPMVTQMAMTDTTPFGQQRVFKEKVDTRTQEPKEGT	120
ACK57816-Guadeloupe-2006        TKQTGSASSMVNGVVRLLTKPWDVVPMVTQMAMTDTTPFGQQRVFKEKVDTRTQEPKEGT	120
BAD36760-Dominican-2001         TKQTGSASSMVNGVVRLLTKPWDVVPMVTQMAMTDTTPFGQQRVFKEKVDTRTQEPKEGT	120
ACQ44492-VirginIsl-2005         TKQTGSASSMVNGVVRLLTKPWDVVPMVTQMAMTDTTPFGQQRVFKEKVDTRTQEPKEGT	120
ACK57811-FrenchGuiana-2006      TKQTGSASSMVNGVVRLLTKPWDVVPMVTQMAMTDTTPFGQQRVFKEKVDTRTQEPKEGT	120
ACS32033-Jamaica-2007           TKQTGSASSMVNGVVRLLTKPWDVVPMVTQMAMTDTTPFGQQRVFKEKVDTRTQEPKEGT	120
ACK57806-Suriname-2005          TKQTGSASSMVNGVVRLLTKPWDVVPMVTQMAMTDTTPFGQQRVFKEKVDTRTQEPKEGT	120
AAW31412-Cuba-1997              TKQTGSASSMVNGVVRLLTKPWDVVPMVTQMAMTDTTPFGQQRVFKEKVDTRTQEPKEGT	120
AET43238-Venezuela-2007         TKQTGSASSMVNGVVRLLTKPWDVVPMVTQMAMTDTTPFGQQRVFKEKVDTRTQEPKEGT	120
ACQ44500-Belize-2002            TKQTGSASSMVNGVVRLLTKPWDVVPMVTQMAMTDTTPFGQQRVFKEKVDTRTQEPKEGT	120
ACW82937-Mexico-2006            TKQTGSASSMVNGVVRLLTKPWDVVPMVTQMAMTDTTPFGQQRVFKEKVDTRTQEPKEGT	120
AHG23135-Nicaragua-2007         TKQTGSASSMVNGVVRLLTKPWDVVPMVTQMAMTDTTPFGQQRVFKEKVDTRTQEPKEGT	120
AGX15366-Peru-2009              TKQTGSASSMVNGVVRLLTKPWDVVPMVTQMAMTDTTPFGQQRVFKEKVDTRTQEPKEGT	120
AGN94892-Brazil-2002            TKQTGSASSMVNGVVRLLTKPWDVVPMVTQMAMTDTTPFGQQRVFKEKVDTRTQEPKEGT	120
AHG23133-PuertoRico-2006        TKQTGSASSMVNGVVKLLTKPWDVVPMVTQMAMTDTTPFGQQRVFKEKVDTRTQEPKEGT	120
ADK26435-Guam-2001              TKQTGSASSMVNGVVRLLTKPWDVIPMVTQMAMTDTTPFGQQRVFKEKVDTRTQEPKEGT	120
AGF87124-China-2012             TKQTGSASSMVNGVVKLLTKPWDVIPMVTQMAMTDTTPFGQQRVFKEKVDTRTQEPKEGT	120
ABW06614-Brunei-2006            TKQTGSASSMVNGVVRLLTKPWDVIPMVTQMAMTDTTPFGQQRVFKEKVDTRTQEPKEGT	120
AAW51407-Jakarta-2004           TKQTGSASSMVNGVVRLLTKPWDVIPMVTQMAMTDTTPFGQQRVFKEKVDTRTQEPKEGT	120
AKA55069-Singapore-2012         TKQTGSASSMVNGVVRLLTKPWDVIPMVTQMAMTDTTPFGQQRVFKEKVDTRTQEPKEGT	120
BAD42415-Sumatera-1998          TKQTGSASSMVNGVVRLLTKPWDVIPMVTQMAMTDTTPFGQQRVFKEKVDTRTQEPKEGT	120
AHG06340-Makassar-2007          TKQTGSASSMVNGVVRLLTKPWDVIPMVTQMAMTDTTPFGQQRVFKEKVDTRTQEPKEGT	120
AAK67712-Australia-1993         TKQTGSASSMVNGVVRLLTKPWDIIPMVTQMAMTDTTPFGQQRVFKEKVDTRTQEPKEGT	120
AHG23169-Cambodia-2008          TKQTGSASSMVNGVVRLLTKPWDVLPTVTQMAMTDTTPFGQQRVFKEKVDTRTQEPKEGT	120
ACY70785-Thailand-2001          TKQTGSASSMVNGVVRLLTKPWDVLPTVTQMAMTDTTPFGQQRVFKEKVDTRTQEPKEGT	120
AGO67249-VietNam-1995           TKQTGSASSMVNGVVRLLTKPWDVVPMVTQMAMTDTTPFGQQRVFKEKVDTRTQEPKEGT	120
ACQ44517-PapuaNewGuinea-2008    TKQTGSASSMVNGVVRLLTKPWDVVPMVTQMAMTDTTPFGQQRVFKEKVDTRTQEPKEGT	120
AHZ61501-Taiwan-2014            TKQTGSASSMVNGVVRLLTKPWDVVPMVTQMAMTDTTPFGQQRVFKEKVDTRTQEPKEGT	120
                                : .******:*****:*******::* ***:********************* :** **

DENV4_AEW50183-Brazil-2010      RMVMTTTANWLWALLGKKKNPRLCTREEFISKVRSNAAIGAVFQEEQGWTSASEAVNDSR	180
DENV4_AFX65868-Brazil-2010      RMVMTTTANWLWALLGKKKNPRLCTREEFISKVRSNAAIGAVFQEEQGWTSASEAVNDSR	180
AHA80987-Pakistan-2010          KKLMKITAEWLWKELGKKKTPRMCTREEFARKVRSNAALGAIFTDENKWKSAREAVEDSR	180
AFZ40225-India-2009             KKLMKITAEWLWKELGKKKTPRMCTREEFTRKVRSNAALGAIFTDENKWKSAREAVEDSR	180
AIH13924-Jeddah-2014            KKLMKITAEWLWKELGKKKTPRMCTREEFARKVRSNAALGAIFTDENKWKSAREAVEDSR	180
ACS32039-SriLanka-2004          KKLMKITAEWLWKELRKKKTPRMCTREEFARKVRSNAALGAIFTDENKWKSAREAVEDSR	180
ADM26228-Tahiti-1971            KKLMKITAEWLWKELGKKKTPRMCTREEFTKKVRSNAALGAIFTDENKWKSXREAVEDSR	180
ADM26226-Samoa-1972             KKLMKITAEWLWKELGKKKTPRMCTREEFTKKVRSNAALGAIFTDENKWKSAREAVEDSR	180
ADM26236-Tonga-1974             KKLMKITAEWLWKELGKKKTPRMCTREEFTKKVRSNAALGAIFTDENKWKSAREAVEDSR	180
ADM26220-Fiji-1971              KKLMKITAEWLWKELGKKKTPRMCTREEFTKKVRSNAALGAIFTDENKWKSAREAVEDSR	180
ADM26222-NewCaledonia-1972      KKLMKITAEWLWKELGKKKTPRMCTREEFTKKVRSNAALGAIFTDENKWKSAREAVEDSR	180
ACQ44499-SaintKitts-2001        KKLMKITAEWLWKELGKKKTPRMCTREEFTKKVRSNAALGAIFTDENKWKSAREAVEDGR	180
AER45462-Guatemala-2009         KKLMKITAEWLWKELGKKKTPRMCTREEFTRKVRSNAALGAIFTDENKWKSAREAVEDGR	180
ADA60759-Colombia-2007          KKLMKITAEWLWKELGKKKTPRMCTREEFTRKVRSNAALGAIFTDENKWKSAREAVEDGR	180
ACK57816-Guadeloupe-2006        KKLMKITAEWLWKELGKKKTPRMCTREEFTRKVRSNAALGAIFTDENKWKSAREAVEDGR	180
BAD36760-Dominican-2001         KKLMKITAEWLWKELGKEKTPRMCTREEFTRKVRSNAALGAIFTDENKWKSAREAVEDGR	180
ACQ44492-VirginIsl-2005         KKLMKITAEWLWKELGKEKTPRMCTREEFTRKVRSNAALGAIFTDENKWKSAREAVEDGR	180
ACK57811-FrenchGuiana-2006      KKLMKITAEWLWKELGKEKTPRMCTREEFTRKVRSNAALGAVFTDENKWKSAREAVEDGR	180
ACS32033-Jamaica-2007           KKLMKITAEWLWKELGKEKTPRMCTREEFTRKVRSNAALGAIFTDENKWKSAREAVEDGR	180
ACK57806-Suriname-2005          KKLMKITAEWLWKELGKEKTPRMCTREEFTRKVRSNAALGAIFTDENKWKSAREAVEDGR	180
AAW31412-Cuba-1997              KKLMKITAEWLWKELGKKKTPRMCTREEFTRKVRSNAALGAIFTDENKWKSAREAVEDGR	180
AET43238-Venezuela-2007         KKLMRITAEWLWKELGKKKTPRMCTREEFTRKVRSNAALGAIFTDENKWKSAREAVEDGR	180
ACQ44500-Belize-2002            KKLMKITAEWLWKELGKKKTPRMCTREEFTRKVRSNAALGAIFTDENKWKSAREAVEDGR	180
ACW82937-Mexico-2006            KKLMKITAEWLWKELGKKKTPRMCTREEFTRKVRSNAALGAIFTDENKWKSAREAVEDGR	180
AHG23135-Nicaragua-2007         KKLMKITAEWLWKELGKKKTPRMCTREEFTKKVRSNAALGAIFTDENKWKSAREAVEDGR	180
AGX15366-Peru-2009              KKLMKITAEWLWKELGKKKTPRMCTREEFTRKVRSNAALGAIFTDENKWKSAREAVEDSR	180
AGN94892-Brazil-2002            KKLMRITAEWLWKELGKKKTPRMCTREEFTRKVRSNAALGAIFTDENKWKSAREAVEDSR	180
AHG23133-PuertoRico-2006        KKLMRITAEWLWKELGKKKTPRMCTREEFTRKVRSNAALGAIFTDENKWKSAREAVEDSR	180
ADK26435-Guam-2001              KKLMKITAEWLWKELGKKKTPRMCTREEFTRKVRSNAALGAIFTDENKWKSAREAVEDSG	180
AGF87124-China-2012             KKLMKITAEWLWKELGKKKTPRMCTREEFTRKVRSNAALGAIFTDENKWKSARGAVEDSG	180
ABW06614-Brunei-2006            KKLMKITAEWLWKELGKKKTPRMCTREEFTRKVRSNAALGAIFTDENKWKSAREAVEDSG	180
AAW51407-Jakarta-2004           KKLMKITAEWLWKELGKKKTPRMCTREEFTRKVRSNAALGAIFTDENKWKSAREAVEDSE	180
AKA55069-Singapore-2012         KKLMKITAEWLWKELGKKKTPRMCTREEFTRKVRSNAALGAIFTDENKWKSAREAVEDSG	180
BAD42415-Sumatera-1998          KKLMKITAEWLWKELGKKKTPRMCTREEFTRKVRSNAALGAIFTDENKWKSAREAVEDSG	180
AHG06340-Makassar-2007          KKLMKITAEWLWKELGKKKTPRMCTREEFTRKVRSNAALGAIFTDENKWKSAREAVEDSG	180
AAK67712-Australia-1993         KKLMKITAEWLWKELGKKKTPRMCTREEFTRKVRSNAALGAIFTDENKWKSAREAVEDSG	180
AHG23169-Cambodia-2008          KKLMKITAEWLWKELGKKKTPRMCTREEFTRKVRSNAALGAIFTDENKWKSAREAVEDSR	180
ACY70785-Thailand-2001          KKLMKITAEWLWKELGKKKTPRMCTREEFTRKVRSNAALGAIFTDENKWKSAREAVEDSR	180
AGO67249-VietNam-1995           KKLMKITAEWLWKELGKKKTPRMCTREEFTRKVRSNAALGAIFTDENKWKSAREAVEDSR	180
ACQ44517-PapuaNewGuinea-2008    KKLMKITAEWLWKELGKKKTPRMCTREEFTRKVRSNAALGAIFTDENKWKSAREAVEDSR	180
AHZ61501-Taiwan-2014            KKLMKITAEWLWKELGKKKTPRMCTREEFTRKVRSNAALGAIFTDENKWKSAREAVEDSR	180
                                : :*   **:***  * *:*.**:******  *******:**:* :*: *.*   **:*.

DENV4_AEW50183-Brazil-2010      FWELVDKERALHQEGKCESCVYNMMGKREKKLGEFGRAKGSRAIWYMWLGARFLEFEALG	240
DENV4_AFX65868-Brazil-2010      FWELVDKERALHQEGKCESCVYNMMGKREKKLGEFGRAKGSRAIWYMWLGARFLEFEALG	240
AHA80987-Pakistan-2010          FWELVDKERNLHLEGKCETCVYNMMGKREKKIGEFGKAKGSRAVWYMWLGARFLEFEALG	240
AFZ40225-India-2009             FWELVNKERNLHLEGKCETCVYNMMGKREKKIGEFGKAKGSRAIWYMWLGARFLEFEALG	240
AIH13924-Jeddah-2014            FWELVDKERNLHLEGKCETCVYNMMGKREKKIGEFGKAKGSRAIWYMWLGARFLEFEALG	240
ACS32039-SriLanka-2004          FWELVDKERNLHLEGKCETCVYNMMGKREKKIGEFGKAKGSRAIWYMWLGARFLEFEALG	240
ADM26228-Tahiti-1971            FWELVDKERNLHLEGKCETCVYNMMGKREKKLGEFGKAKGSRAIWYMWLGARFLEFEALG	240
ADM26226-Samoa-1972             FWELVDKERNLHLEGKCETCVYNMMGKREKKLGEFGKAKGSRAIWYMWLGARFLEFEALG	240
ADM26236-Tonga-1974             FWELVDKERNLHLEGKCETCVYNMMGKREKKLGEFGKAKGSRAIWYMWLGARFLEFEALG	240
ADM26220-Fiji-1971              FWELVDKERNLHLEGKCETCVYNMMGKREKKLGEFGKAKGSRAIWYMWLGARFLEFEALG	240
ADM26222-NewCaledonia-1972      FWELVDKERNLHLEGKCETCVYNMMGKREKKLGEFGKAKGSRAIWYMWLGARFLEFEALG	240
ACQ44499-SaintKitts-2001        FWELVDRERNLHLEGKCETCVYNMMGKREKKLGEFGKAKGSRAIWYMWLGARFLEFEALG	240
AER45462-Guatemala-2009         FWELVDRERNLHLEGKCETCVYNMMGKREKKLGEFGKAKGSRAIWYMWLGARFLEFEALG	240
ADA60759-Colombia-2007          FWELVDRERNLHLEGKCETCVYNMMGKREKKLGEFGKAKGSRAIWYMWLGARFLEFEALG	240
ACK57816-Guadeloupe-2006        FWELVDRERNLHLEGKCETCVYNMMGKREKKLGEFGKAKGSRAIWYMWLGARFLEFEALG	240
BAD36760-Dominican-2001         FWELVDRERNLHLEGKCETCVYNMMGKREKKLGEFGKAKGSRAIWYMWLGARFLEFEALG	240
ACQ44492-VirginIsl-2005         FWELVDRERNLHLEGKCETCVYNMMGKREKKLGEFGKAKGSRAIWYMWLGARFLEFEALG	240
ACK57811-FrenchGuiana-2006      FWELVDRERNLHLEGKCETCVYNMMGKREKKLGEFGKAKGSRAIWYMWLGARFLEFEALG	240
ACS32033-Jamaica-2007           FWELVDRERNLHLEGKCETCVYNMMGKREKKLGEFGKAKGSRAIWYMWLGARFLEFEALG	240
ACK57806-Suriname-2005          FWELVDRERNLHLEGKCETCVYNMMGKREKKLGEFGKAKGSRAIWYMWLGARFLEFEALG	240
AAW31412-Cuba-1997              FWELVDRERNLHLEGKCETCVYNMMGKREKKLGEFGKAKGSRAIWYMWLGARFLEFEALG	240
AET43238-Venezuela-2007         FWELVDRERNLHLEGKCETCVYNMMGKREKKLGEFGKAKGSRAIWYMWLGARFLEFEALG	240
ACQ44500-Belize-2002            FWELVDRERNLHLEGKCETCVYNMMGKREKKLGEFGKAKGSRAIWYMWLGARFLEFEALG	240
ACW82937-Mexico-2006            FWELVDRERNLHLEGKCETCVYNMMGKREKKLGEFGKAKGSRAIWYMWLGARFLEFEALG	240
AHG23135-Nicaragua-2007         FWELVDRERNLHLEGKCETCVYNMMGKREKKLGEFGKAKGSRAIWYMWLGARFLEFEALG	240
AGX15366-Peru-2009              FWELVDRERNLHLEGKCETCVYNMMGKREKKLGEFGKAKGSRAIWYMWLGARFLEFEALG	240
AGN94892-Brazil-2002            FWELVDRERNLHLEGKCETCVYNMMGKREKKLGEFGKAKGSRAIWYMWLGARFLEFEALG	240
AHG23133-PuertoRico-2006        FWELVDRERNLHLEGKCETCVYNMMGKREKKLGEFGKAKGSRAIWYMWLGARFLEFEALG	240
ADK26435-Guam-2001              FWELVDKERNLHLEGKCETCVYNMMGKREKKLGEFGKAKGSRAIWYMWLGARFLEFEALG	240
AGF87124-China-2012             FWELVDKERNLHLEGKCETCVYNMMGKREKKLGEFGKAKGSRAIWYMWLGARFLEFEALG	240
ABW06614-Brunei-2006            FWELVDKERNLHLEGKCETCVYNMMGKREKKLGEFGKAKGSRAIWYMWLGARFLEFEALG	240
AAW51407-Jakarta-2004           FWELVDKERNLHLEGKCETCVYNMMGKREKKLGEFGKAKGSRAIWYMWLGARFLEFEALG	240
AKA55069-Singapore-2012         FWELVDKERNLHLEGKCETCVYNMMGKREKKLGEFGKAKGSRAIWYMWLGARFLEFEALG	240
BAD42415-Sumatera-1998          FWELVDKERNLHLEGKCETCVYNMMGKREKKLGEFGKAKGSRAIWYMWLGARFLEFEALG	240
AHG06340-Makassar-2007          FWELVDKERNLHLEGKCETCVYNMMGKREKKLGEFGKAKGSRAIWYMWLGARFLEFEALG	240
AAK67712-Australia-1993         FWELVDKERNLHLEGKCETCVYNMMGKREKKLGEFGKAKGSRAIWYMWLGARFLEFEALG	240
AHG23169-Cambodia-2008          FWELVDKERNLHLEGKCETCVYNMMGKREKKLGEFGKAKGSRAIWYMWLGARFLEFEALG	240
ACY70785-Thailand-2001          FWELVDKERNLHLEGKCETCVYNMMGKREKKLGEFGKAKGSRAIWYMWLGARFLEFEALG	240
AGO67249-VietNam-1995           FWELVDKERNLHLEGKCETCVYNMMGKREKKLGEFGKAKGSRAIWYMWLGARFLEFEALG	240
ACQ44517-PapuaNewGuinea-2008    FWELVDKERNLHLEGKCETCVYNMMGKREKKLGEFGKAKGSRAIWYMWLGARFLEFEALG	240
AHZ61501-Taiwan-2014            FWGLVDKERNLHLEGKCETCVYNMMGKREKKLGEFGKAKGSRAIWYMWLGARFLEFEALG	240
                                ** **::** ** *****:************:****:******:****************

DENV4_AEW50183-Brazil-2010      FLNEDHWFGRENSWSGVEGEGLHRLGYILEEIDKKDGDLMYADDTAGWDTRITEDDLQNE	300
DENV4_AFX65868-Brazil-2010      FLNEDHWFGRENSWSGVEGEGLHRLGYILEEIDKKDGDLMYADDTAGWDTRITEDDLQNE	300
AHA80987-Pakistan-2010          FLNEDHWFSRENSLSGVEGEGLHKLGYILREVSKKEGGAMYADDTAGWDTRITLEDLKNE	300
AFZ40225-India-2009             FLNEDHWFSRENSLSGVEGEGLHKLGYILREVSKKEGGAMYADDTAGWDTRITLEDLKNE	300
AIH13924-Jeddah-2014            FLNEDHWFSRENSLSGVEGEGLHKLGYILREVSKKEGGAMYADDTAGWDTRITLEDLKNE	300
ACS32039-SriLanka-2004          FLNEDHWFSRENSLSGVEGEGLHKLGYILREVSKKEGGAMYADDTAGWDTRITLEDLKNE	300
ADM26228-Tahiti-1971            FLNEDHWFSRENSLSGVEGEGLHKLGYILREVSKKEGGAMYADDTAGWDTRITIEDLKNE	300
ADM26226-Samoa-1972             FLNEDHWFSRENSLSGVEGEGLHKLGYILREVSKKEGGAMYADDTAGWDTRITIEDLKNE	300
ADM26236-Tonga-1974             FLNEDHWFSRENSLSGVEGEGLHKLGYILREVXKKEGGAMYADDTAGWDTRITIEDLKNE	300
ADM26220-Fiji-1971              FLNEDHWFSRENSLSGVEGEGLHKLGYILREVSKKEGGAMYADDTAGWDTRITIEDLKNE	300
ADM26222-NewCaledonia-1972      FLNEDHWFSRENSLSGVEGEGLHKLGYILREVSKKEGGAMYADDTAGWDTRITIEDLKNE	300
ACQ44499-SaintKitts-2001        FLNEDHWFSRGNSLSGVEGEGLHRLGYILREVGKKEGGAMYADDTAGWDTRITLEDLKNE	300
AER45462-Guatemala-2009         FLNEDHWFSRGNSLSGVEGEGLHRLGYILREVSKKEGGAMYADDTAGWDTRITLEDLKNE	300
ADA60759-Colombia-2007          FLNEDHWFSRGNSLSGVEGEGLHRLGYILRDVSKKEGGAMYADDTAGWDTRITLEDLKNE	300
ACK57816-Guadeloupe-2006        FLNEDHWFSRGNSLSGVEGEGLHRLGYILREVGKKEGGAMYADXTAGWDTRITLEDLKNE	300
BAD36760-Dominican-2001         FLNEDHWFSRGNSLSGVEGEGLHRLGYILREVGKKEGGAMYADDTAGWDTRITLEDLKNE	300
ACQ44492-VirginIsl-2005         FLNEDHWFSRGNSLSGVEGEGLHRLGYILREVGKKEGGAMYADDTAGWDTRITLEDLKNE	300
ACK57811-FrenchGuiana-2006      FLNEDHWFSRGNSLSGVEGEGLHRLGYILREVGKKEGGAMYADDTAGWDTRITLEDLKNE	300
ACS32033-Jamaica-2007           FLNEDHWFSRGNSLSGVEGEGLHRLGYILREVGKKEGGAMYADDTAGWDTRITLEDLKNE	300
ACK57806-Suriname-2005          FLNEDHWFSRGNSLSGVEGEGLHRLGYILREVGKKEGGAMYADDTAGWDTRITLEDLKNE	300
AAW31412-Cuba-1997              FLNEDHWFSRGNSLSGVEGEGLHRLGYILREVGKKEGGAMYADDTAGWDTRITLEDLKNE	300
AET43238-Venezuela-2007         FLNEDHWFSRGNSLSGVEGEGLHRLGYILRDVGKKEGGAMYADDTAGWDTRITLEDLKNE	300
ACQ44500-Belize-2002            FLNEDHWFSRGNSLSGVEGEGLHRLGYILRDVGKKEGGAMYADDTAGWDTRITLEDLKNE	300
ACW82937-Mexico-2006            FLNEDHWFSRGNSLSGVEGEGLHRLGYILRDVGKKEGGAMYADDTAGWDTRITLEDLKNE	300
AHG23135-Nicaragua-2007         FLNEDHWFSRGNSLSGVEGEGLHRLGYILRDVGKKEGGAMYADDTAGWDTRITLEDLKNE	300
AGX15366-Peru-2009              FLNEDHWFSRGNSLSGVEGEGLHRLGYILRDVSKKEGGAMYADDTAGWDTRITLEDLKNE	300
AGN94892-Brazil-2002            FLNEDHWFSRGNSLSGVEGEGLHRLGYILRDVSKKEGGAMYADDTAGWDTRITLEDLKNE	300
AHG23133-PuertoRico-2006        FLNEDHWFSRGNSLSGVEGEGLHKLGYILRDVSKKEGGAMYADDTAGWDTRITLEDLKNE	300
ADK26435-Guam-2001              FLNEDHWFSRKNSLSGVEGEGLHKLGYILRDVSKKEGGAMYADDTAGWDTRITLEDLKNE	300
AGF87124-China-2012             FLNEDHWFSRENSLSGVEGEGLHKLGYILRDVSKKEGGAMYADDTAGWDTRITLEDLKNE	300
ABW06614-Brunei-2006            FLNEDHWFSRENSLSGVEGEGLHKLGYILRDVSKKEGGAMYADDTAGWDTRITLEDLKNE	300
AAW51407-Jakarta-2004           FLNEDHWFSRENSLSGVEGEGLHKLGYILRDVSKKEGGAMYADDTAGWDTRITLEDLKNE	300
AKA55069-Singapore-2012         FLNEDHWFSRENSLSGVEGEGLHKLGYILRDVSKKEGGAMYADDTAGWDTRITLEDLKNE	300
BAD42415-Sumatera-1998          FLNEDHWFSRENSLSGVEGEGLHKLGYILRDVSKKEGGAMYADDTAGWDTRITLEDLKNE	300
AHG06340-Makassar-2007          FLNEDHWFSRENSLSGVEGEGLHKLGYILRDVSKKEGGAMYADDTAGWDTRITLEDLKNE	300
AAK67712-Australia-1993         FLNEDHWFSRENSLSGVEGEGLHKLGYILRDVSKKEGGAMYADDTAGWDTRITLEDLKNE	300
AHG23169-Cambodia-2008          FLNEDHWFSRENSLSGVEGEGLHKLGYILRDVSKKEGGAMYADDTAGWDTRITLEDLKNE	300
ACY70785-Thailand-2001          FLNEDHWFSRENSLSGVEGEGLHKLGYILRDVSKKEGGAMYADDTAGWDTRITLEDLKNE	300
AGO67249-VietNam-1995           FLNEDHWFSRENSLSGVEGEGLHKLGYILRDVSKKEGGAMYADDTAGWDTRITLEDLKNE	300
ACQ44517-PapuaNewGuinea-2008    FLNEDHWFSRENSLSGVEGEGLHKLGYILRDVSKKEGGAMYADDTAGWDTRITLEDLKNE	300
AHZ61501-Taiwan-2014            FLNEDHWFSRENSLSGVEGEGLHKLGYILRDVSKKEGGAMYADDTAGWDTRITLEDLKNE	300
                                ********.* ** *********:*****.:: **:*. **** ********* :**:**

DENV4_AEW50183-Brazil-2010      ELITEQMAPHHKILAKAIFKLTYQNKVVKVLRPTPRGAVMDIISRKDQRGSGQVGTYGLN	360
DENV4_AFX65868-Brazil-2010      ELITEQMAPHHKILAKAIFKLTYQNKVVKVLRPTPRGAVMDIISRKDQRGSGQVGTYGLN	360
AHA80987-Pakistan-2010          EMVTNHMEGEHKKLAEAIFKLTYQNKVVRVQRPTPRGTVMDIISRRDQRSSGQVGTYGLN	360
AFZ40225-India-2009             EMVTNHMEGEHKKLAEAIFKLTYQNKVVRVQRPTPRGTVMDIISRRDQRGSGQVGTYGLN	360
AIH13924-Jeddah-2014            EMVTNHMEGEHKKLAEAIFKLTYQNKVVRVQRPTPRGTVMDIISRRDQRGSGQVGTYGLN	360
ACS32039-SriLanka-2004          EMVTNHMEGEHKKLAEAIFKLTYQNKVVRVQRPTPRGTVMDIISRRDQRGSGQVGTYGLN	360
ADM26228-Tahiti-1971            EMITNHMAGEHKKLAEAIFKLTYQNKVVRVQRPTPRGTVMDIISRRDQRGSGQVGTYGLN	360
ADM26226-Samoa-1972             EMITNHMAGEHKKLAEAIFKLTYQNKVVRVQRPTPRGTVMDIISRRDQRGSGQVGTYGLN	360
ADM26236-Tonga-1974             EMITNHMAGEHKKLAEAIFKLTYQNKVVRVQRPTPRGTVMDIISRRDQRGSGQVGTYGLN	360
ADM26220-Fiji-1971              EMITNHMAGEHKKLAEAIFKLTYQNKVVRVQRPTPRGTVMDIISRRDQRGSGQVGTYGLN	360
ADM26222-NewCaledonia-1972      EMITNHMAGEHKKLAEAIFKLTYQNKVVRVQRPTPRGTVMDIISRRDQRGSGQVGTYGLN	360
ACQ44499-SaintKitts-2001        EMVTNHMKGEHKKLAEAIFKLTYQNKVVRVQRPTPRGTVMDIISRRDQRGSGQVGTYGLN	360
AER45462-Guatemala-2009         EMVTNHMKGEHKKLAEAIFKLTYQNKVVRVQRPTPRGTVMDIISRRDQRGSGQVGTYGLN	360
ADA60759-Colombia-2007          EMVTNHMKGEHKKLAEAIFKLTYQNKVVRVQRPTPRGTVMDIISRRDQRGSGQVGTYGLN	360
ACK57816-Guadeloupe-2006        EMVTNHMKGEHKKLAEAIFKLTYQNKVVRVQRPTPRGTVMDIISRKDQRGSGQVGTYGLN	360
BAD36760-Dominican-2001         EMVTNHMKGEHKKLAEAIFKLTYQNKVVRVQRPTPRGTVMDIISRKDQRGSGQVGTYGLN	360
ACQ44492-VirginIsl-2005         EMVTNHMKGEHKKLAEAIFKLTYQNKVVRVQRPTPRGTVMDIISRKDQRGSGQVGTYGLN	360
ACK57811-FrenchGuiana-2006      EMVTNHMKGEHKKLAEAIFKLTYQNKVVRVQRPTPRGTVMDIISRKDQRGSGQVGTYGLN	360
ACS32033-Jamaica-2007           EMVTNHMKGEHKKLAEAIFKLTYQNKVVRVQRPTPRGTVMDIISRKDQRGSGQVGTYGLN	360
ACK57806-Suriname-2005          EMVTNHMKGEHKKLAEAIFKLTYQNKVVRVQRPTPRGTVMDIISRKDQRGSGQVGTYGLN	360
AAW31412-Cuba-1997              EMVTNHMKGEHKKLAEAIFKLTYQNKVVRVQRPTPRGTVMDIISRRDQRGSGQVGTYGLN	360
AET43238-Venezuela-2007         EMVTNHMKGEHKKLAEAIFKLTYQNKVVRVQRPTPRGTVMDIISRRDQRGSGQVGTYGLN	360
ACQ44500-Belize-2002            EMVTNHMKGEHKKLAEAIFKLTYQNKVVRVQRPTPRGTVMDIISRRDQRGSGQVGTYGLN	360
ACW82937-Mexico-2006            EMVTNHMKGEHKKLAEAIFKLTYQNKVVRVQRPTPRGTVMDIISRRDQRGSGQVGTYGLN	360
AHG23135-Nicaragua-2007         EMVTNHMKGEHKKLAEAIFKLTYQNKVVRVQRPTPRGTVMDIISRRDQRGSGQVGTYGLN	360
AGX15366-Peru-2009              EMVTNHMKGEHKKLAEAIFKLTYQNKVVRVQRPTPRGTVMDIISRRDQRGSGQVGTYGLN	360
AGN94892-Brazil-2002            EMVTNHMEGEHKKLAEAIFKLTYQNKVVRVQRPTPRGTVMDIISRKDQRGSGQVGTYGLN	360
AHG23133-PuertoRico-2006        EMVTNHMKGEHKKLAEAIFKLTYQNKVVRVQRPTPRGTVMDIISRKDQRGSGQVGTYGLN	360
ADK26435-Guam-2001              EMVTNHMEGEHKKLAEAIFRLTYQNKVVRVQRPTPRGTVMDIISRRDQRGSGQVVTYGLN	360
AGF87124-China-2012             EMVTNHMEGVHKKLAEAIFRLTYQNKVVRVQRPTPRGTVMDIISRRDQRGSGQVVTYGLN	360
ABW06614-Brunei-2006            EMVTNHMEGEHKKLAEAIFKLTYQNKVVRVQRPTPRGTVMDIISRRDQRGSGQVVTYGLN	360
AAW51407-Jakarta-2004           EMVTNHMEGEHKKLAEAIFRLTYQNKVVRVQRPTPRGTVMDIISRRDQRGSGQVVTYGLN	360
AKA55069-Singapore-2012         EMVTNHMEGEHKKLAEAIFKLTYQNKVVRVQRPTPRGTVMDIISRRDQRGSGQVVTYGLN	360
BAD42415-Sumatera-1998          EMVTNHMEGEHKKLAEAIFKLTYQNKVVRVQRPTPRGTVMDIISRRDQRGSGQVVTYGLN	360
AHG06340-Makassar-2007          EMVTNHMEGEHKKLAEAIFKLTYQNKVVRVQRPTPRGTVMDIISRRDQRGSGQVVTYGLN	360
AAK67712-Australia-1993         EMVTNHMEGEHKKLAEAIFKLTYQNKVVRVQRPTPRGTVMDIISRRDQRGSGQVVTYGLN	360
AHG23169-Cambodia-2008          EMVTNHMEGEHKKLAEAIFKLTYQNKVVRVQRPTPRGTVMDIISRRDQRGSGQVGTYGLN	360
ACY70785-Thailand-2001          EMVTNHMEGEHKKLAEAIFKLTYQNKVVRVQRPTPRGTVMDIISRRDQRGSGQVGTYGLN	360
AGO67249-VietNam-1995           EMVTNHMEGEHKKLAEAIFKLTYQNKVVRVQRPTPRGTVMDIISRRDQRGSGQVGTYGLN	360
ACQ44517-PapuaNewGuinea-2008    EMVTNHMEGEHKKLAEAIFKLTYQNKVVRVQRPTPRGTVMDIISRRDQRGSGQVGTYGLN	360
AHZ61501-Taiwan-2014            EMVTNHMEGEHKKLAEAIFKLTYQNKVVRVQRPTPRGTVMDIISRRDQRGSGQVGTYGLN	360
                                *::*::*   ** **:***:********:* ******:*******:***.**** *****

DENV4_AEW50183-Brazil-2010      TFTNMEVQLIRQMEAEGVITQDDMQNPKGLKERVEKWLRECGVDRLKRMAISGDDCVVKP	420
DENV4_AFX65868-Brazil-2010      TFTNMEVQLIRQMEAEGVITQDDMQNPKGLKERVEKWLRECGVDRLKRMAISGDDCVVKP	420
AHA80987-Pakistan-2010          TFTNMGAQLIRQMEGEGVFKGIQHLT-ATEEVAVQDWLARVGRERLSRMAISGDDCVVKP	419
AFZ40225-India-2009             TFTNMGAQLIRQMEGEGVFKGIQHLT-ATEETAVHDWLARVGRERLSRMAISGDDCVVKP	419
AIH13924-Jeddah-2014            TFTNMGAQLIRQMEGEGVFKGIQHLT-ATEEVAVQDWLARVGRERLSRMAISGDDCVVKP	419
ACS32039-SriLanka-2004          TFTNMGAQLIRQMEGEGVFKGIQHLT-ATEEVAVQDWLARVGRERLSRMAISGDDCVVKP	419
ADM26228-Tahiti-1971            TFTNMEAQLIRQMEGEGIFKSIQHLT-ASEEIAVQDWLVRVGRERLSRMAISGDDCVVKP	419
ADM26226-Samoa-1972             TFTNMEAQLIRQMEGEGIFKSIQHLT-ASEEIAVQDWLVRVGRERLSRMAISGDDCVVKP	419
ADM26236-Tonga-1974             TFTNMEAQLIRQMEGEGIFKSIQHLT-ASEEIAVQDWLVRVGRERLSRMAISGDDCVVKP	419
ADM26220-Fiji-1971              TFTNMEAQLIRQMEGEGIFKSIQHLT-ASEEIAVQDWLVRVGRERLSRMAISGDDCVVKP	419
ADM26222-NewCaledonia-1972      TFTNMEAQLIRQMEGEGIFKSIQHLT-ASEEIAVQDWLVRVGRERLSRMAISGDDCVVKP	419
ACQ44499-SaintKitts-2001        TFTNMEAQLIRQMEGEGIFKSIQHLT-ATEEIAVQNWLARVGRERLSRMAISGDDCVVKP	419
AER45462-Guatemala-2009         TFTNMEAQLIRQMEGEGIFKSIQHLT-ATEEIAVQNWLARVGRERLSRMAISGDDCVVKP	419
ADA60759-Colombia-2007          TFTNMEAQLIRQMEGEGIFKSIQHLT-AIEEIAVQNWLARVGRERLSRMAISGDDCVVKP	419
ACK57816-Guadeloupe-2006        TFTNMEAQLIRQMEGEGIFKSIQHLT-ATEEIAVQNWLARVGRERLSRMAISGDDCVVKP	419
BAD36760-Dominican-2001         TFTNMEAQLIRQMEGEGIFKSIQHLT-ATEEIAVQNWLARVGRERLSRMAISGDDCVVKP	419
ACQ44492-VirginIsl-2005         TFTNMEAQLIRQMEGEGIFKSIQHLT-VTEEIAVQNWLARVGRERLSRMAISGDDCVVKP	419
ACK57811-FrenchGuiana-2006      TFTNMEAQLIRQMEGEGIFKSIQHLT-ATEEIAVQNWLARVGRERLSRMAISGDDCVVKP	419
ACS32033-Jamaica-2007           TFTNMEAQLIRQMEGEGIFKSIQHLT-ATEEIAVQNWLARVGRERLSRMAISGDDCVVKP	419
ACK57806-Suriname-2005          TFTNMEAQLIRQMEGEGIFKSIQHLT-ATEEIAVQNWLARVGRERLSRMAISGDDCVVKP	419
AAW31412-Cuba-1997              TFTNMEAQLIRQMEGEGIFKSIQHLT-ATEEIAVQNWLARVGRERLSRMAISGDDCVVKP	419
AET43238-Venezuela-2007         TFTNMEAQLIRQMEGEGIFKSIQHLT-VTEEIAVQNWLARVGRERLSRMAISGDDCVVKP	419
ACQ44500-Belize-2002            TFTNMEAQLIRQMEGEGIFKSIQHLT-ATEEIAVQNWLARLGRERLSRMAISGDDCVVKP	419
ACW82937-Mexico-2006            TFTNMEAQLIRQMEGEGIFKSIQHLT-ATEEIAVQNWLARVGRERLSRMAISGDDCVVKP	419
AHG23135-Nicaragua-2007         TFTNMEAQLIRQMEGEGIFKSIQHLT-ATEEIAVQNWLARVGRERLSRMAISGDDCVVKP	419
AGX15366-Peru-2009              TFTNMEAQLIRQMEGEGIFKSIQHLT-VTEEIAVQNWLARVGRERLSRMAISGDDCVVKP	419
AGN94892-Brazil-2002            TFTNMEAQLIRQMEGEGIFKSIQHLT-VTEEIAVQNWLARVGRERLSRMAISGDDCVVKP	419
AHG23133-PuertoRico-2006        TFTNMEAQLIRQMEGEGIFKSIQRLT-VTEEIAVQNWLARVGRERLSRMAISGDDCVVKP	419
ADK26435-Guam-2001              TFTNMEAQLIRQMEGEGVFKSIQHLT-ATEEIAVKTWLVRVGRERLSRMAISGDDCVVKP	419
AGF87124-China-2012             TFTNMEAQLIRQMEGEGVFKSIQHLT-VTEEIAVKNWLVRVGRERLSRMAISGDDCVVKP	419
ABW06614-Brunei-2006            TFTNMEAQLIRQMEGEGVFKSIQQLT-VTEEIAVKNWLARVGRERLSRMAISGDDCVVKP	419
AAW51407-Jakarta-2004           TFTNMEAQLIRQMEGEGVFKSIQHLT-VTEEIAVKNWLVRVGRERLSRMAISGDDCVVKP	419
AKA55069-Singapore-2012         TFTNMEAQLIRQMEGEGVFKSIQQLT-VKEEIAVKNWLIRVGRERLSRMAISGDDCVVKP	419
BAD42415-Sumatera-1998          TFTNMEAQLIRQMEGEGVFKSIQHLT-VTEEIAVKNWLARVGRERLSRMAISGDDCVVKP	419
AHG06340-Makassar-2007          TFTNMEAQLIRQMEGEGVFKSIQQLT-VTEEIAVKNWLVRVGRERLSRMAISGDDCVVKP	419
AAK67712-Australia-1993         TFTNMEAQLIRQMEGEGVFKSIQHLT-VTEEIAVKNWLVRVGRERLSRMAISGDDCVVKP	419
AHG23169-Cambodia-2008          TFTNMEAQLIRQMEGEGVFKNIQHLT-VTEEAAVQNWLARVGRERLSRMAISGDDCVVKP	419
ACY70785-Thailand-2001          TFTNMEAQLIRQMEGEGVFKNIQHLT-VTEEVAVQNWLARVGRERLSRMAISGDDCVVKP	419
AGO67249-VietNam-1995           TFTNMEAQLIRQMEGEGIFKSIQHLT-VTEEIAVQDWLARVGRERLSRMAISGDDCVVKP	419
ACQ44517-PapuaNewGuinea-2008    TFTNMEAQLIRQMEGEGVFKSIQHLT-VTEEIAVQNWLARVGRERLSRMAISGDDCVVKP	419
AHZ61501-Taiwan-2014            TFTNMEAQLIRQMEGEGVFKSIQHLT-VTEEIAVQNWLARVGRERLSRMAISGDDCVVKP	419
                                ***** .*******.**::.  :  .     :  *. ** . * :**.*************

DENV4_AEW50183-Brazil-2010      LDERFGTSLLFLNDMGKVRKDIPQWEPSKGWKNWQEVPFCSHHFHKIFMKDGRSLVVPCR	480
DENV4_AFX65868-Brazil-2010      LDERFGTSLLFLNDMGKVRKDIPQWEPSKGWKNWQEVPFCSHHFHKIFMKDGRSLVVPCR	480
AHA80987-Pakistan-2010          LDDRFASALTALNDMGKVRKDIQQWEPSRGWNDWTQVPFCSHHFHELIMKDGRVLVVPCR	479
AFZ40225-India-2009             LDDRFASALTALNDMGKVRKDIQQWEPSRGWNDWTQVPFCSHHFHELIMKDGRVLVVPCR	479
AIH13924-Jeddah-2014            LDDRFASALTALNDMGKVRKDIQQWEPSRGWNDWTQVPFCSHHFHELIMKDGRVLVVPCR	479
ACS32039-SriLanka-2004          LDDRFASALTALNDMGKVRKDIQQWEPSRGWNDWTQVPFCSHHFHELIMKDGRVLVVPCR	479
ADM26228-Tahiti-1971            LDDRFARALTALNDMGKVRKDIQQWEPSRGWNDWTQVPFCSHHFHELIMKDGRVLVVPCR	479
ADM26226-Samoa-1972             LDDRFARALTALNDMGKVRKDIQQWEPSRGWNDWTQVPFCSHHFHELIMKDGRTLVVPCR	479
ADM26236-Tonga-1974             LDDRFARALTALNDMGKVRKDIQQWEPSRGWNDWTQVPFCSHHFHELIMKDGRTLVVPCR	479
ADM26220-Fiji-1971              LDDRFARALTALNDMGKVRKDIQQWEPSRGWNDWTQVPFCSHHFHELIMKDGRTLVVPCR	479
ADM26222-NewCaledonia-1972      LDDRFARALTALNDMGKVRKDIQQWEPSRGWNDWTQVPFCSHHFHELIMKDGRTLVVPCR	479
ACQ44499-SaintKitts-2001        IDDRFASALTALNDMGKVRKDIQQWEPSRGWNDWTQVPFCSHHFHELVMKDGRVLVVPCR	479
AER45462-Guatemala-2009         IDDRFASALTALNDMGKVRKDIQQWEPSRGWNDWTQVPFCSHHFHELVMKDGRVLVVPCR	479
ADA60759-Colombia-2007          IDDRFASALTALNDMGKVRKDIQQWEPSRGWNDWTQVPFCSHHFHELVMKDGRVLVVPCR	479
ACK57816-Guadeloupe-2006        IDDRFASALTALNDMGKVRKDIQQWEPSRGWNDWTQVPFCSHHFHELVMKDGRVLVVPCR	479
BAD36760-Dominican-2001         IDDRFASALTALNDMGKVRKDIQQWEPSRGWNDWTQVPFCSHHFHELVMKDGRVLVVPCR	479
ACQ44492-VirginIsl-2005         IDDRFASALTALNDMGKVRKDIQQWEPSRGWNDWTQVPFCSHHFHELVMKDGRVLVVPCR	479
ACK57811-FrenchGuiana-2006      IDDRFASALTALNDMGKVRKDIQQWEPSRGWNDWTQVPFCSHHFHELVMKDGRVLVVPCR	479
ACS32033-Jamaica-2007           IDDRFASALTALNDMGKVRKDIQQWEPSRGWNDWTQVPFCSHHFHELVMKDGRVLVVPCR	479
ACK57806-Suriname-2005          IDDRFASALTALNDMGKVRKDIQQWEPSRGWNDWTQVPFCSHHFHELVMKDGRVLVVPCR	479
AAW31412-Cuba-1997              IDDRFASALTALNDMGKVRKDIQQWEPSRGWNDWTQVPFCSHHFHELVMKDGRVLVVPCR	479
AET43238-Venezuela-2007         IDDRFASALTALNDMGKVRKDIQQWEPSRGWNDWTQVPFCSHHFHELVMKDGRVLVVPCR	479
ACQ44500-Belize-2002            IDDRFASALTALNDMGKVRKDIQQWEPSRGWNDWTQVPFCSHHFHELVMKDGRVLVVPCR	479
ACW82937-Mexico-2006            IDDRFASALTALNDMGKVRKDIQQWEPSRGWNDWTQVPFCSHHFHELVMKDGRVLVVPCR	479
AHG23135-Nicaragua-2007         IDDRFASALTALNDMGKVRKDIQQWEPSRGWNDWTQVPFCSHHFHELVMKDGRVLVVPCR	479
AGX15366-Peru-2009              LDDRFASALTALNDMGKVRKDIQQWEPSRGWNDWTQVPFCSHHFHELVMKDGRVLVVPCR	479
AGN94892-Brazil-2002            LDDRFASALTALNDMGKVRKDIQQWEPSRGWNDWTQVPFCSHHFHELVMKDGRVLVVPCR	479
AHG23133-PuertoRico-2006        LDDRFASALTALNDMGKVRKDIQQWEPSRGWNDWTQVPFCSHHFHELVMKDGRVLVVPCR	479
ADK26435-Guam-2001              LDDRFASALTALNDMGKVRKDIQQWEPSRGWNDWTQVPFCSHHFHELIMKDGRVLVVPCR	479
AGF87124-China-2012             LDDRFASALTALNDMGKVRKDIQQWEPSRGWNDWTQVPFCSHHFHELIMKDGRVLVVPCR	479
ABW06614-Brunei-2006            LDDRFASALTALNDMGKVRKDIQQWEPSRGWNDWTQVPFCSHHFHELIMKDGRVLVVPCR	479
AAW51407-Jakarta-2004           LDDRFASALTALNDMGKVRKDIQQWEPSRGWNDWTQVPFCSHHFHELIMKDGRVLVVPCR	479
AKA55069-Singapore-2012         LDDRFASALTALNDMGKVRKDIQQWEPSRGWNDWTQVPFCSHHFHELIMKDGRVLVVPCR	479
BAD42415-Sumatera-1998          LDDRFASALTALNDMGKVRKDIQQWEPSRGWNDWTQVPFCSHHFHELIMKDGRVLVVPCR	479
AHG06340-Makassar-2007          LDDRFASALTALNDMGKVRKDIQQWEPSRGWNDWTQVPFCSHHFHELIMKDGRVLVVPCR	479
AAK67712-Australia-1993         LDDRFASALTALNDMGKVRKDIQQWEPSRGWNDWTQVPFCSHHFHELIMKDGRVLVVPCR	479
AHG23169-Cambodia-2008          LDDRFANALTALNDMGKIRKDIQQWEPSKGWNDWTQVPFCSHHFHELIMKDGRVLVVPCR	479
ACY70785-Thailand-2001          LDDRFANALTALNDMGKIRKDIQQWEPSKGWNDWTQVPFCSHHFHELIMKDGRVLVVPCR	479
AGO67249-VietNam-1995           LDDRFASALTALNDMGKVRKDIQQWEPSRGWNDWTQVPFCSHHFHELIMKDGRVLVVPCR	479
ACQ44517-PapuaNewGuinea-2008    LDDRFASALTALNDMGKVRKDIQQWEPSRGWNDWTQVPFCSHHFHELIMKDGRVLVVPCR	479
AHZ61501-Taiwan-2014            LDDRFASALTALNDMGKVRKDIQQWEPSRGWSDWTQVPFCSHHFHELIMKDGRVLVVPCR	479
                                :*:**. :*  ******:**** *****:**.:* :*********::.***** ******

DENV4_AEW50183-Brazil-2010      NQDELIGRARISQGAGWSLRETACLGKAYAQMWSLMYFHRRDLRLASMAICSAVPTEWFP	540
DENV4_AFX65868-Brazil-2010      NQDELIGRARISQGAGWSLRETACLGKAYAQMWSLMYFHRRDLRLASMAICSAVPTEWFP	540
AHA80987-Pakistan-2010          NQDELIGRARISQGAGWSLRETACLGKSYAQMWSLMYFHRRGLRLAANAICSAVPSHWVP	539
AFZ40225-India-2009             NQDELIGRARISQGAGWSLRETACLGKSYAQMWSLMYFHRRDLRLAANAICSAVPSHWVP	539
AIH13924-Jeddah-2014            NQDELIGRARISQGAGWSLRETACLGKSYAQMWSLMYFHRRDLRLAANAICSAVPSHWVP	539
ACS32039-SriLanka-2004          NQDELIGRARISQGAGWSLRETACLGKSYAQMWSLMYFHRRDLRLAANAICSAVPSHWVP	539
ADM26228-Tahiti-1971            NQDELIGRARISQGAGWSLRETACLGKSYAQMWSLMYFHRRDLRLAANAICSAVPSHWIP	539
ADM26226-Samoa-1972             NQDELIGRARISQGAGWSLRETACLGKSYAQMWSLMYFHRRDLRLAANAICSAVPPHWIP	539
ADM26236-Tonga-1974             NQDELIGRARISQGAGWSLRETACLGKSYAQMWSLMYFHRRDLRLAANAICSAVPSHWIP	539
ADM26220-Fiji-1971              NQDELIGRARISQGAGWSLRETACLGKSYAQMWSLMYFHRRDLRLAANAICSAVPSHWIP	539
ADM26222-NewCaledonia-1972      NQDELIGRARISQGAGWSLRETACLGKSYAQMWSLMYFHRRDLRLAANAICSAVPSHWIP	539
ACQ44499-SaintKitts-2001        NQDELIGRARISQGAGWSLKETACLGKSYAQMWTLMYFHRRDLRLAANAICSAVPSHWVP	539
AER45462-Guatemala-2009         NQDELIGRARISQGAGWSLKETACLGKSYAQMWTLMYFHRRDLRLAANAICSAVPSHWVP	539
ADA60759-Colombia-2007          NQDELIGRARISQGAGWSLKETACLGKSYAQMWTLMYFHRRDLRLAANAICSAVPSHWVP	539
ACK57816-Guadeloupe-2006        NQDELIGRARISQGAGWSLKETACLGKSYAQMWTLMYFHRRDLRLAANAICSAVPSHWVP	539
BAD36760-Dominican-2001         NQDELIGRARISQGAGWSLKETACLGKSYAQMWTLMYFHRRDLRLAANAICSAVPSHWVP	539
ACQ44492-VirginIsl-2005         NQDELIGRARISQGAGWSLKETACLGKSYAQMWTLMYFHRRDLRLAANAICSAVPSHWVP	539
ACK57811-FrenchGuiana-2006      NQDELIGRARISQGAGWSLKETACLGKSYAQMWTLMYFHRRDLRLAANAICSAVPSHWVP	539
ACS32033-Jamaica-2007           NQDELIGRARISQGAGWSLKETACLGKSYAQMWTLMYFHRRDLRLAANAICSAVPSHWVP	539
ACK57806-Suriname-2005          NQDELIGRARISQGAGWSLKETACLGKSYAQMWTLMYFHRRDLRLAANAICSAVPSHWVP	539
AAW31412-Cuba-1997              SQDELIGRARISQGAGWSLKETACLGKSYAQMWTLMYFHRRDLRLAANAICSAVPSHWVP	539
AET43238-Venezuela-2007         NQDELIGRARISQGAGWSLKETACLGKSYAQMWTLMYFHRRDLRLAANAICSAVPSHWVP	539
ACQ44500-Belize-2002            NQDELIGRARISQGAGWSLKETACLGKSYAQMWTLMYFHRRDLRLAANAICSAVPSHWVP	539
ACW82937-Mexico-2006            NQDELIGRARISQGAGWSLKETACLGKSYAQMWTLMYFHRRDLRLAANAICSAVPSHWVP	539
AHG23135-Nicaragua-2007         NQDELIGRARISQGAGWSLKETACLGKSYAQMWTLMYFHRRDLRLAANAICSAVPSHWVP	539
AGX15366-Peru-2009              NQDELIGRARISQGAGWSLKETACLGKSYAQMWTLMYFHRRDLRLAANAICSAVPSHWVP	539
AGN94892-Brazil-2002            NQDELIGRARISQGAGWSLKETACLGKSYAQMWTLMYFHRRDLRLAANAICSAVPSHWVP	539
AHG23133-PuertoRico-2006        NQDELIGRARISQGAGWSLKETACLGKSYAQMWTLMYFHRRDLRLAANAICSAVPSHWVP	539
ADK26435-Guam-2001              NQDELIGRARISQGAGWSLRETACLGKSYAQMWSLMYFHRRDLRLAANAICSAVPSHWVP	539
AGF87124-China-2012             NQDELIGRARISQGAGWSLRETACLGKSYAQMWSLMYFHRRDLRLAANAICSAVPSHWVP	539
ABW06614-Brunei-2006            NQDELIGRARISQGAGWSMRETACLGKSYAQMWSLMYFHRRDLRLAANAICSAVPSHWVP	539
AAW51407-Jakarta-2004           NQDELIGRARISQGAGWSLRETACLGKSYAQMWSLMYFHRRDLRLAANAICSAVPSHWVP	539
AKA55069-Singapore-2012         NQDELIGRARISQGAGWSLRETACLGKSYAQMWSLMYFHRRDLRLAANAICSAVPSHWIP	539
BAD42415-Sumatera-1998          NQDELIGRARISQGAGWSLRETACLGKSYAQMWSLMYFHRRDLRLAANAICSAVPSHWVP	539
AHG06340-Makassar-2007          NQDELIGRARISQGAGWSLRETACLGKSYAQMWSLMYFHRRDLRLAANAICSAVPSHWVP	539
AAK67712-Australia-1993         NQDELIGRARISQGAGWSLRETACLGKSYAQMWSLMYFHRRDLRLAANAICSAVPSHWVP	539
AHG23169-Cambodia-2008          NQDELIGRARISQGAGWSLRETACLGKSYAQMWSLMYFHRRDLRLAANAICSAVPSHWVP	539
ACY70785-Thailand-2001          NQDELIGRARISQGAGWSLRETACLGKSYAQMWSLMYFHRRDLRLAANAICSAVPSHWVP	539
AGO67249-VietNam-1995           NQDELIGRARISQGAGWSLKETACLGKSYAQMWSLMYFHRRDLRLAANAICSAVPSHWVP	539
ACQ44517-PapuaNewGuinea-2008    NQDELIGRARISQGAGWSLRETACLGKSYAQMWSLMYFHRRDLRLAANAICSAVPSHWVP	539
AHZ61501-Taiwan-2014            NQDELIGRARISQGAGWSLRETACLGKSYAQMWSLMYFHRRDLRLAANAICSAVPSHWVP	539
                                .****************::*******:*****:*******.****: ******* .*.*

DENV4_AEW50183-Brazil-2010      TSRTTWSIHAHHQWMTTEDMLKVWNRVWIEDNPNMIDKTPVHSWEDIPYLGKREDLWCGS	600
DENV4_AFX65868-Brazil-2010      TSRTTWSIHAHHQWMTTEDMLKVWNRVWIEDNPNMIDKTPVHSWEDIPYLGKREDLWCGS	600
AHA80987-Pakistan-2010          TSRTTWSIHARHEWMTTEDMLTVWNRVWIQENPWMEDKTPVESWEEIPYLGKRGDQWCGS	599
AFZ40225-India-2009             TSRTTWSIHARHEWMTTEDMLTVWNRVWIQENPWMEDKTPVESWEEIPYLGKREDQWCGS	599
AIH13924-Jeddah-2014            TSRTTWSIHAGHEWMTTEDMLTVWNRVWIQENPWMEDKTPVESWEEIPYLGKREDQWCGS	599
ACS32039-SriLanka-2004          TSRTTWSIHARHEWMTTEDMLTVWNRVWIQENPWMEDKTPVESWEEIPYLGKREDQWCGS	599
ADM26228-Tahiti-1971            TSRTTWSIHASHEWMTTEDMLTVWNRVWILENPWMEDKTPVESWEEIPYLGKREDQWCGS	599
ADM26226-Samoa-1972             TSRTTWSIHASHEWMTTEDMLTVWNRVWILENPWMEDKTPVESWEEIPYLGKREDQWCGS	599
ADM26236-Tonga-1974             TSRTTWSIHASHEWMTTEDMLTVWNRVWILENPWMEDKTPVESWEEIPYLGKREDQWCGS	599
ADM26220-Fiji-1971              TSRTTWSIHASHEWMTTEDMLTVWNRVWILENPWMEDKTPVESWEEIPYLGKREDQWCGS	599
ADM26222-NewCaledonia-1972      TSRTTWSIHASHEWMTTEDMLTVWNRVWILENPWMEDKTPVESWEEIPYLGKREDQWCGS	599
ACQ44499-SaintKitts-2001        TSRTTWSIHAKHEWMTTEDMLAVWNRVWIQENPWMEDKTPVESWEEVPYLGKREDQWCGS	599
AER45462-Guatemala-2009         TSRTTWSIHAKHEWMTTEDMLAVWNRVWIQENPWMEDKTPVESWEEVPYLGKREDQWCGS	599
ADA60759-Colombia-2007          TSRTTWSIHAKHEWMTTEDMLAVWNRVWIQENPWMEDKTPVESWEEVPYLGKREDQWCGS	599
ACK57816-Guadeloupe-2006        TSRTTWSIHAKHEWMTTEDMLAVWNRVWIQENPWMEDKTPVESWEEVPYLGKREDQWCGS	599
BAD36760-Dominican-2001         TSRTTWSIHAKHEWMTTEDMLAVWNRVWIQENPWMEDKTPVESWEEVPYLGKREDQWCGS	599
ACQ44492-VirginIsl-2005         TSRTTWSIHAKHEWMTTEDMLAVWNRVWIQENPWMEDKTPVESWEEVPYLGKREDQWCGS	599
ACK57811-FrenchGuiana-2006      TSRTTWSIHAKHEWMTTEDMLAVWNRVWIQENPWMEDKTPVESWEEVPYLGKREDQWCGS	599
ACS32033-Jamaica-2007           TSRTTWSIHAKHEWMTTEDMLAVWNRVWIQENPWMEDKTPVESWEEVPYLGKREDQWCGS	599
ACK57806-Suriname-2005          TSRTTWSIHAKHEWMTTEDMLAVWNRVWIQENPWMEDKTPVESWEEVPYLGKREDQWCGS	599
AAW31412-Cuba-1997              TSRTTWSIHAKHEWMTTEDMLAVWNRVWIQENPWMEDKTPVESWEEVPYLGKREDQWCGS	599
AET43238-Venezuela-2007         TSRTTWSIHAKHEWMTTEDMLAVWNRVWIQENPWMEDKTPVESWEEVPYLGKREDQWCGS	599
ACQ44500-Belize-2002            TSRTTWSIHAKHEWMTTEDMLAVWNRVWIQENPWMEDKTPVESWEEVPYLGKREDQWCGS	599
ACW82937-Mexico-2006            TSRTTWSIHAKHEWMTTEDMLAVWNRVWIQENPWMEDKTPVESWEEVPYLGKREDQWCGS	599
AHG23135-Nicaragua-2007         TSRTTWSIHAKHEWMTTEDMLAVWNRVWIQENPWMEDKTPVESWEEVPYLGKREDQWCGS	599
AGX15366-Peru-2009              TSRTTWSIHAKHEWMTTEDMLAVWNRVWIQENPWMEDKTPVESWEEVPYLGKREDQWCGS	599
AGN94892-Brazil-2002            TSRTTWSIHAKHEWMTTEDMLAVWNRVWIQENPWMEDKTPVESWEEVPYLGKREDQWCGS	599
AHG23133-PuertoRico-2006        TSRTTWSIHAKHEWMTTEDMLAVWNRVWIQENPWMEDKTPVESWEEVPYLGKREDQWCGS	599
ADK26435-Guam-2001              TSRTTWSIHATHEWMTTEDMLTVWNRVWIQENPWMEDKTPVESWEEIPYLGKREDQWCGS	599
AGF87124-China-2012             TSRTTWSIHATHEWMTTEDMLTVWNRVWIQENPWMEDKTPVESWEEIPYLGKREDQWCGS	599
ABW06614-Brunei-2006            TSRTTWSIHATHEWMTTEDMLTVWNRVWIQENPWMEDKTPVESWEEIPYLGKREDQWCGS	599
AAW51407-Jakarta-2004           TSRTTWSIHATHEWMTTEDMLTVWNRVWIQENPWMEDKTPVESWEEIPYLGKREDQWCGS	599
AKA55069-Singapore-2012         TSRTTWSIHATHEWMTTEDMLTVWNRVWIQENPWMEDKTPVESWEEIPYLGKREDQWCGS	599
BAD42415-Sumatera-1998          TSRTTWSIHATHEWMTTEDMLTVWNRVWIQENPWMEDKTPVESWEEIPYLGKREDQWCGS	599
AHG06340-Makassar-2007          TSRTTWSIHATHEWMTTEDMLTVWNRVWIQENPWMEDKTPVESWEEIPYLGKREDQWCGS	599
AAK67712-Australia-1993         TSRTTWSIHATHEWMTTEDMLTVWNRVWIQENPWMEDKTPVESWEEIPYLGKREDQWCGS	599
AHG23169-Cambodia-2008          TSRTTWSIHAKHEWMTTEDMLTVWNRVWIRENPWMEDKTPVESWEEIPYLGKREDQWCGS	599
ACY70785-Thailand-2001          TSRTTWSIHAKHEWMTTEDMLTVWNRVWIRENPWMEDKTPVESWEEIPYLGKREDQWCGS	599
AGO67249-VietNam-1995           TSRTTWSIHAKHEWMTTEDMLTVWNRVWIQENPWMEDKTPVESWEEVPYLGKREDQWCGS	599
ACQ44517-PapuaNewGuinea-2008    TSRTTWSIHAKHEWMTTEDMLTVWNRVWIQENPWMEDKTPVESWEEIPYLGKREDQWCGS	599
AHZ61501-Taiwan-2014            TSRTTWSIHAKHEWMTTEDMLTVWNRVWIQENPWMEDKTPVESWEEIPYLGKREDQWCGS	599
                                ********** *:******** ******* :** * *****.***::****** * ****

DENV4_AEW50183-Brazil-2010      LIGLSSRATWAKNIHTAITQVRNLIGKEEYVDYMPVMKR-	639
DENV4_AFX65868-Brazil-2010      LIGLSSRATWAKNIHTAITQVRNLIGKEEYVDYMPVMKR-	639
AHA80987-Pakistan-2010          LIGLTSRATWARNIQTAINRVRSLIGSEGYTDYMPSMKRF	639
AFZ40225-India-2009             LIGLTSRATWAKNIQTAINQVRSLIGSEEYTDYMPSMKRF	639
AIH13924-Jeddah-2014            LIGLTSRATWARNIQTAINQVRSLIGSEEYTDYMPSMKRF	639
ACS32039-SriLanka-2004          LIGLTSRATWAKNIQTAINQVRSLIGSEEYTDYMPSMKRF	639
ADM26228-Tahiti-1971            LIGLTSRATWAKNIQTAINQVRSLIGNEEYTDYMPSMKRF	639
ADM26226-Samoa-1972             LIGLTSRATWAKNIQTAINQVRSLIGNEEYTDYMPSMKRF	639
ADM26236-Tonga-1974             LIGLTSRATWAKNIQTAINQVRSLIGNEEYTDYMPSMKRF	639
ADM26220-Fiji-1971              LIGLTSRATWAKNIQTAINQVRSLIGNEEYTDYMPSMKRF	639
ADM26222-NewCaledonia-1972      LIGLTSRATWAKNIQTAINQVRSLIGNEEYTDYMPSMKRF	639
ACQ44499-SaintKitts-2001        LIGLTSRATWAKNIQTAINQVRSLIGNEEYTDYMPSMKRF	639
AER45462-Guatemala-2009         LIGLTSRATWAKNIQTAINQVRSLIGNEEYTDYMPSMKRF	639
ADA60759-Colombia-2007          LIGLTSRATWAKNIQTAINQVRSLIGNEEYTDYMPSMKRF	639
ACK57816-Guadeloupe-2006        LIGLTSRATWAKNIQTAINQVRSLIGNEEYTDYMPSMKRF	639
BAD36760-Dominican-2001         LIGLTSRATWAKNIQTAINQVRSLIGNEEYTDYMPSMKRF	639
ACQ44492-VirginIsl-2005         LIGLTSRATWAKNIQTAINQVRSLIGNEEYTDYMPSMKRF	639
ACK57811-FrenchGuiana-2006      LIGLTSRATWAKNIQTAINQVRSLIGNEGYTDYMPSMKRF	639
ACS32033-Jamaica-2007           LIGLTSRATWAKNIQTAINQVRSLIGNEGYTDYMPSMKRF	639
ACK57806-Suriname-2005          LIGLTSRATWAKNIQTAINQVRSLIGNEGYTDYMPSMKRF	639
AAW31412-Cuba-1997              LIGLTSRATWAKNIQTAINQVRSLIGNEEYTDYMPSMKRF	639
AET43238-Venezuela-2007         LIGLTSRATWAKNIQTAINQVRSLIGNEEYTDYMPSMKRF	639
ACQ44500-Belize-2002            LIGLTSRATWAKNIQTAINQVRSLIGNEEYTDYMPSMKRF	639
ACW82937-Mexico-2006            LIGLTSRATWAKNIQTAINQVRSLIGNEEYTDYMPSMKRF	639
AHG23135-Nicaragua-2007         LIGLTSRATWAKNIQTAINQVRSLIGNEEYTDYMPSMKRF	639
AGX15366-Peru-2009              LIGLTSRATWAKNIKTAINQVRSLMGNEEYMDYMPSMKRF	639
AGN94892-Brazil-2002            LIGLTSRATWAKNIQTAINQVRSLIGNEEYTDYMPSMKRF	639
AHG23133-PuertoRico-2006        LIGLTSRATWAKNIQTAINQVRSLIGNEEYTDYMPSMKRF	639
ADK26435-Guam-2001              LIGLTSRATWAKNIQTAINQVRSLIGNEEYTDYMPSMKRF	639
AGF87124-China-2012             LIGLTSRATWAKNIQTAINQVRSLIGNEEYTDYMPSMKRF	639
ABW06614-Brunei-2006            LIGLTSRATWAKNIQTAINQVRSLIGNEEYTDYMPSMKRF	639
AAW51407-Jakarta-2004           LIGLTSRATWAKNIQTAINQVRSLIGNEEYTDYMPSMKRF	639
AKA55069-Singapore-2012         LIGLTSRATWAKNIQTAINQVRSLIGNEEYTDYMPSMKRF	639
BAD42415-Sumatera-1998          LIGLTSRATWAKNIQTAINQVRSLIGNEEYTDYMPSMKRF	639
AHG06340-Makassar-2007          LIGLTSRATWAKNIQTAINQVRSLIGNEEYTDYMPSMKRF	639
AAK67712-Australia-1993         LIGLTSRATWAKNIQTAINQVRSLIGNEEYTDYMPSMKRF	639
AHG23169-Cambodia-2008          LIGLTSRATWAKNIQAAINQVRSLIGNEEYTDYMPSMKRF	639
ACY70785-Thailand-2001          LIGLTSRATWAKNIQAAINQVRSLIGNEEYTDYMPSMKRF	639
AGO67249-VietNam-1995           LIGLTSRATWAKNIQTAINQVRSLIGNEEYTDYMPSMKRF	639
ACQ44517-PapuaNewGuinea-2008    LIGLTSRATWAKNIQTAINQVRSLIGNEEYTDYMPSMKRF	639
AHZ61501-Taiwan-2014            LIGLTSRATWAKNIQTAINQVRSLIGNEEYTDYMPSMKRF	639
                                ****:******:**::**.:**.*:*.* * **** ***
```

PLEASE NOTE: Showing colors on large alignments is slow.

This website requires cookies, and the limited processing of your personal data in order to function. By using the site you are agreeing to this as outlined in our Privacy Notice and Terms of Use.

I agree, dismiss this banner
